# The Phosphate Transporter PiT1 (Slc20a1) Revealed As a New Essential Gene for Mouse Liver Development

**DOI:** 10.1371/journal.pone.0009148

**Published:** 2010-02-10

**Authors:** Laurent Beck, Christine Leroy, Sarah Beck-Cormier, Anne Forand, Christine Salaün, Nadine Paris, Adeline Bernier, Pablo Ureña-Torres, Dominique Prié, Mario Ollero, Laure Coulombel, Gérard Friedlander

**Affiliations:** 1 INSERM, U845, Centre de Recherche Croissance et Signalisation, Paris, France; 2 Faculté de Médecine, Université Paris Descartes, Paris, France; 3 Unité de Génétique Fonctionnelle de la Souris, Institut Pasteur, Paris, France; 4 Unité de Génétique Fonctionnelle de la Souris, CNRS, URA2578, Paris, France; 5 INSERM, U935, Villejuif, France; 6 Hôpital Paul Brousse, Villejuif, France; University of Florida, United States of America

## Abstract

**Background:**

*PiT1* (or *SLC20a1*) encodes a widely expressed plasma membrane protein functioning as a high-affinity Na^+^-phosphate (Pi) cotransporter. As such, PiT1 is often considered as a ubiquitous supplier of Pi for cellular needs regardless of the lack of experimental data. Although the importance of PiT1 in mineralizing processes have been demonstrated *in vitro* in osteoblasts, chondrocytes and vascular smooth muscle cells, *in vivo* evidence is missing.

**Methodology/Principal Findings:**

To determine the *in vivo* function of *PiT1*, we generated an allelic series of *PiT1* mutations in mice by combination of wild-type, hypomorphic and null *PiT1* alleles expressing from 100% to 0% of *PiT1*. In this report we show that complete deletion of *PiT1* results in embryonic lethality at E12.5. *PiT1*-deficient embryos display severely hypoplastic fetal livers and subsequent reduced hematopoiesis resulting in embryonic death from anemia. We show that the anemia is not due to placental, yolk sac or vascular defects and that hematopoietic progenitors have no cell-autonomous defects in proliferation and differentiation. In contrast, mutant fetal livers display decreased proliferation and massive apoptosis. Animals carrying two copies of hypomorphic *PiT1* alleles (resulting in 15% *PiT1* expression comparing to wild-type animals) survive at birth but are growth-retarded and anemic. The combination of both hypomorphic and null alleles in heterozygous compounds results in late embryonic lethality (E14.5–E16.5) with phenotypic features intermediate between null and hypomorphic mice. In the three mouse lines generated we could not evidence defects in early skeleton formation.

**Conclusion/Significance:**

This work is the first to illustrate a specific *in vivo* role for *PiT1* by uncovering it as being a critical gene for normal developmental liver growth.

## Introduction

PiT1 is a membrane protein originally identified as the human retrovirus receptor for GALV (Gibbon Ape Leukemia Virus), and was therefore designated GLVR1 [1]. Based on the observation that GLVR1 possesses sequence similarity to *pho-4^+^*, a phosphate (Pi) permease of *Neurospora crassa*, several groups have demonstrated that GLVR1 is a high affinity Na^+^-dependent Pi transporter, which was consequently renamed PiT1 [2], [3], [4]. PiT1 belongs to the Pi transporter (PiT) family comprising conserved symporters throughout all kingdoms that use either sodium or proton gradients to transport Pi [5]. In mammals, the PiT family is assigned to the solute carrier series SLC20 which also includes PiT2, initially identified as the amphotropic murine retrovirus receptor Ram-1 [6] and subsequently characterized as a Pi transporter with transport characteristics very close to PiT1 [2], [4]. The broad tissue distribution of PiT1 and PiT2 has led to the proposal that these transporters could play a housekeeping role for cellular Pi homeostasis [2], [7], [8]. Although plausible, direct experimental data is lacking. Moreover, significant differences between PiT1 and PiT2 expression levels and ratios are observed among tissues [9], which suggests that these two transporters may have specific and non redundant roles *in vivo*.

Due to the requirement of large amounts of Pi in bone physiology, the regulation of PiT1 has been well documented in bone and cartilage *in vitro* models. In osteoblast-like cells, *PiT1*, but not *PiT2*, mRNA expression and Na^+^-Pi cotransport are regulated by various factors such as Pi [10], epinephrine [11], insulin-like growth factor 1 (IGF-1) [12], [13] and bone morphogenic protein 2 (BMP2) [14]. Importantly, PiT1, but not PiT2 is upregulated during osteoblast differentiation, consistent with a dedicated role of PiT1 in this process [15]. Expression and activity of PiT1 in chondrogenic cells was found to be regulated by extracellular Pi concentration [16] and transforming growth factor-β (TGF-β) [17]. Moreover, vascular calcification occurring in pathological situations such as hyperphosphatemia-induced calcifications of blood vessels and osteoarthritis, shares a number of similarities with osteogenesis and bone mineralization [18]. Recent *in vitro* studies have suggested that PiT1 may be implicated in pathological vascular calcification [19]. Particularly, inhibition of Pi uptake by PiT1 small hairpin RNA in cultured vascular smooth muscle cells (VSMCs) blocked the expression of Pi-induced osteogenic differentiation markers, Runx2 and osteopontin, indicating that PiT1 might be a major mechanism for controlling vascular calcification and VSMC phenotypic state [18], [19].

However, it should be noted that the above-mentioned studies have been conducted in *in vitro* models and it is not clear which role PiT1 plays in normal bone or vascular physiology. Particularly, the discrete expression of *PiT1* in a subset of hypertrophic chondrocytes late in development [20], its weak expression in osteoblasts and VSMCs [21] together with its low transport capacity [22] make it an unlikely candidate to face the tremendous Pi needs for bone or vascular calcification. Moreover, since most studies aimed at elucidating PiT1 regulation and function were conducted in tissues in which Pi *per se* plays an important role (*i.e.* mineralized tissues), very little information is available for other tissues. Since PiT1 is expressed in numerous non-mineralizing tissues, it is entirely possible that PiT1 possesses regulated tissue-specific roles going beyond a housekeeping Na^+^-Pi transport function. In line with this hypothesis, we have recently identified a new function for PiT1, which is independent of its Na^+^-Pi cotransport activity and is critical for cell proliferation [9]. These recent data show for the first time that PiT1, in addition to its retrovirus receptor and Na^+^-Pi cotransporter functions, modulates cell proliferation *in vitro*. The physiological relevance of this third function is unknown, and further illustrates the need for a wider and more physiological approach to decipher the role of PiT1 *in vivo*.

To provide insights into the physiological role of PiT1 in mice, we established an allelic series of *PiT1* mutations arising from the combination of normal, hypomorphic and null *PiT1* alleles in mice. The new mutant mouse lines described here were used to address the following questions: i) is PiT1 essential for embryo development? ii) are PiT1 and PiT2 physiological roles redundant? iii) is PiT1 essential to skeletal or vessel formation during mouse development? iv) are the growth, development or function of specific organs impaired in the absence of PiT1? In what follows, we provide answers to these questions and report as a key finding that PiT1 deficiency causes mid-gestation embryonic lethality due to an impaired liver development leading to profound anemia.

## Methods

### Animals and Ethics Statement

All mouse models used in the study were on a 129sv/J × C57BL6/J mixed background. Animal care and maintenance were provided through the University Paris Descartes accredited Animal Facility at Necker Faculty of Medicine (Paris). Mice were maintained on rodent laboratory chow (Special Diet Services, Witham, Essex, UK) containing 0.73% calcium, 0.52% phosphate and 600 IU/kg of vitamin D_3_. All procedures were approved by the Animal Care and Use Committee of the University Paris Descartes.

### Derivation of PiT1 Genetically Modified Mice

A BAC clone (RP23-160G19) containing the genomic sequence of mouse *PiT1* was purchased (Invitrogen). Using a recombineering approach [23], we constructed a targeting vector containing *PiT1* 5′- and 3′-homologous arms (4115 bp and 3645 bp, respectively), a neomycin resistance (*neo*) cassette flanked by *frt* sites in intron 5, and a 5′ and 3′ *loxP* sites 532 bp upstream and 934 bp downstream from exon 5, respectively. The final vector containing a diphtheria toxin cassette (*MC1-DTA*) permitting negative selection was verified by sequencing. 129/Sv ES cells were electroporated with 20 µg of linearized targeting vector and selected under G418 treatment. *EcoR*V digested genomic DNA from 336 ES clones was screened by Southern blotting using a *PiT1* 5′ external probe. Nine positive clones were further analyzed using 5′ and 3′ probes after digestion of the DNA with two other restriction enzymes, and four clones with the predicted homologous integration were identified. ES cells were injected into C57BL/6 blastocysts, chimaeric males bred to C57BL/6 females and germ-line transmission of the mutation determined by Southern blot analysis and PCR amplification of tail DNA. Heterozygous offspring (*PiT1^neo/+^* mice) were bred either to *ACTB:FLPe* mice [24] to remove the *neo* cassette, leading to *PiT1^lox/+^* animals, or to *PGK-Cre* mice [25] to produce *PiT1* ^Δ*5/+*^ mice. Genotyping of neonates and embryos was performed by PCR from tail or yolk sac DNA. Primers used for recombineering, probe amplification and genotyping are listed in Table S1.

### Gene Expression

Total RNA was isolated from cells and tissue using NucleoSpin RNA columns (Macherey Nagel). Northern analysis of total RNA (25 µg) and RT-PCR amplification were performed as described previously [26]. Specific primers are listed in Table S1. Real-time PCR was performed using SYBR green chemistry (Thermo Scientific) on an ABI Prism 7700 detection system. The glucuronidase or pinin genes were used as reference genes and expression differences were calculated as described previously [27]. *In situ* hybridization was performed as described [28]. *PiT1* probes (Table S1) were transcribed with SP6 or T7 promoters with the DIG RNA labeling kit (Roche Applied Science). Color reaction was performed by using BM purple and sections were counterstained with hematoxilin.

### Hematopoietic Progenitor Cell Assays

Single-cell suspensions from E12.5 fetal livers were plated in 1 ml 1% methylcellulose in Iscove's modified Dulbecco's medium supplemented with 30% FCS, 1% crystallized BSA, and 100 µM 2-mercaptoethanol in the presence of 2 U/ml EPO, 10 ng/ml IL-3, 20 ng/ml thrombopoietin and 100 ng/ml stem cell factor. Samples were plated in duplicate 35-mm bacterial Petri dishes and incubated at 37°C in a humidified incubator containing 5% CO_2_. Colonies were scored at day 2 for CFU-E, day 7 for CFU-granulocyte-macrophage (CFU-GM) and mature burst-forming unit-erythroid (BFU-E), and days 10 to 12 for CFU-granulocyte, erythroid, monocyte, megakaryocyte (CFU-GEMM).

### Immunohistochemistry and Apoptosis Analysis

Embryos were fixed overnight in 4% buffered formalin. Four-micron sections were produced from paraffin embedded samples and immunohistochemistry was performed according to antibody specification. PECAM-1 (CD-31) was detected in embryos, placental or yolk sac sections using a rat purified anti-CD31 antibody (1/100, clone α-MEC 13.3; BD Pharmigen), as described [29]. Proliferating cells were detected with the anti-Ki67 antibody (AbCys). To perform BrDU labeling, pregnant mice were injected intra-peritoneally with 0.3 ml of 50 mg/ml BrDU (Sigma) in PBS. Embryos were harvested after 1 h and fixed overnight in 4% buffered formalin. After embedding in paraffin, detection of BrdU was performed with anti-BrDU antibody (BD Biosciences). To reveal apoptotic cells, DeadEnd^TM^ Colorimetric TUNEL System (Promega) and immunohistochemistry using anti-activated caspase 3 (Asp175; Cell Signalling) were used according to the manufacter's instructions.

### Partial Hepatectomy

Eight- to twelve-week-old wild-type mice were subjected to sham operation or two-thirds partial hepatectomy between 9 AM and 12 PM, under general anesthesia with inhaled isoflurane (n = 4 for each genotype and time point). Animals were killed at different times after surgery. Sham animals were operated without any liver resection. Livers were snap frozen into liquid nitrogen for mRNA preparations. All experiments were approved by the institutional committee and were in accordance with European guidelines for the care and use of laboratory animals.

### Embryonic Fibroblasts Culture and Analysis

Isolation of MEFs was performed using established procedures and cultured in DMEM supplemented with 10% FBS in 5%CO_2_ and a humidified atmosphere. For growth curves, 20,000 MEFs were seeded in triplicates in 24-well plates. Every day, cells were trypsinized and counted. Pi transport was measured as previously described [30]. Apparent affinity constant (K_m_) and maximal transport rate (V_max_) were calculated by nonlinear curve fitting, assuming Michaelis-Menten kinetics.

### Immunoblot Analysis

Cells were lysed for 30 min in ice-cold lysis buffer (150 mM NaCl, 10 mM Tris HCl, 5 mM EDTA, 1% NP40, 0.1% SDS, 0.5% deoxycholate, 1 mM Na_3_VO_4_, 1 mM NaF, 5 mM Na pyrophosphate, 0.2 M PMSF and a protease inhibitor cocktail from Roche), and the protein extract was boiled and transferred to PVDF membranes. After blocking with 5% milk/TBST, blots were probed with anti-AMPKσ, anti-phospho-AMPKσ (Thr172) antibodies (Cell Signaling Technology) and secondary antibody according to the manufacter's instructions.

### Fatty Acid, Phospholipids and Cholesterol Analysis

The lipid-containing organic phase was obtained from erythrocyte suspensions by liquid-liquid extraction with 6 volumes of chloroform/methanol (2∶1, v/v), centrifuged at 800 g for 3 min, and the resulting lower phase was aspirated. Cholesterol and phospholipid content were monitored by thin layer chromatography. For cholesterol analysis, aliquots of 4 µl of the different extracts and cholesterol standard were applied to HP-K plates (Whatman, Clifton, NJ), developed in chloroform/acetone (95∶5 v/v), stained with the CuSO_4_ reagent, and developed by charring at 170°C. For phospholipid analysis, two sequential mobile phases were utilized as follows: chloroform/triethylamine/ethanol/water (30∶30∶34∶8) and hexane/diethyl ether (100∶4.5). The resulting bands were analyzed by the Imagemaster software (Amersham Biosciences). For fatty acid analysis, a 30 µl aliquot of a 1 mg/ml solution of heptadecanoate (17∶0, Sigma Chemical, St Louis, MO) in chloroform-methanol (1∶1, vol/vol) was added to extracts as an internal standard. Organic extracts were evaporated to dryness under a nitrogen stream and transmethylated with boron trifluoride and methanolic base reagent (BF3-methanol) (Supelco) as previously described [31]. Briefly, dry extracts were resuspended in 0.5 ml of methanolic base, vortexed, and incubated at 100°C for 3 min, followed by addition of 14% BF3 in methanol (0.5 ml), vortexing, incubation at 100°C for 1 min, addition of hexane (0.5 ml), vortexing, incubation at 100°C for 1 min, and addition of 6.5 ml of saturated NaCl. Samples were vortexed and centrifuged at 800 g for 2 min. The hexane upper layer was transferred to a fresh glass tube. Methyl esters of fatty acids were injected into a Hewlett-Packard 5890A gas chromatograph with a flame ionization detector. A 30 m Supelcowax column (Supelco) (0.5 mm internal diameter) was used. The oven temperature was 150°C for 2 min, increased to 200°C for 5 min, held at 200°C for 4 min, increased to 240°C for 8 min, and after 3 min increased to 260°C and held at this temperature until the end of the program. The detector temperature was 300°C. Fatty acid methyl ester peaks were identified by comparison of retention times with a standard mixture of fatty acid methyl esters (Supelco) and quantified by comparison with the internal standard detector response.

### Cartilage and Bone Staining

For staining and visualization of whole skeletons, fetuses or mice were dissected and stained with alizarin red S or alcian blue 8GX (Sigma), or both, as described previously [32].

## Results

### Generation of a *PiT1* Mutant Allelic Series

**Figure 1:**
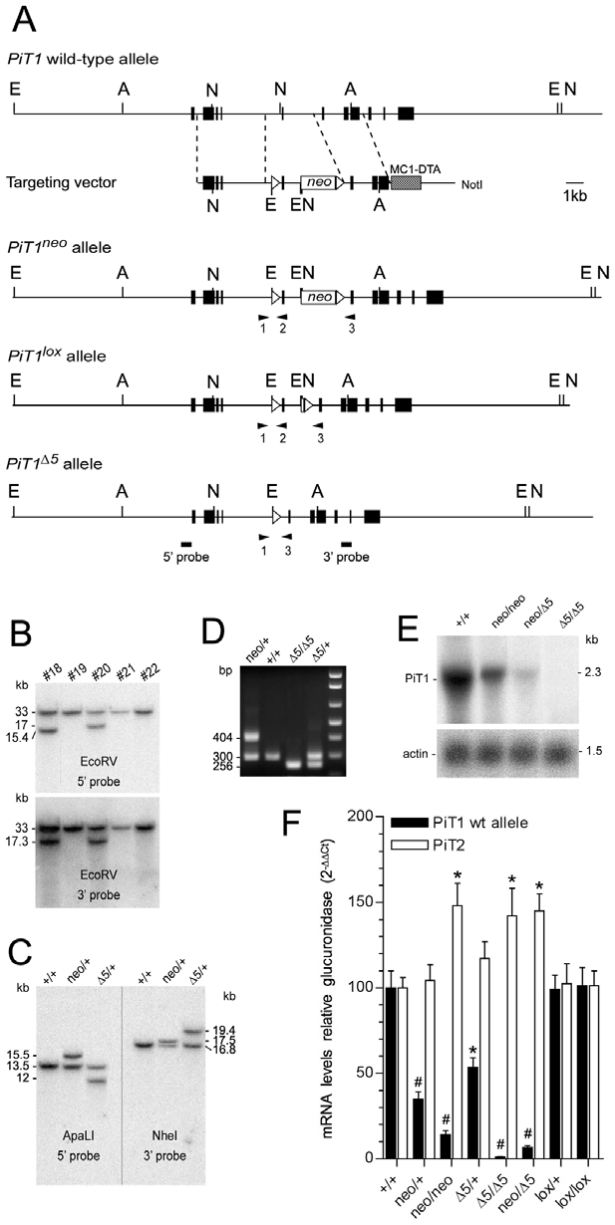
Generation of an allelic series of mutations at the *PiT1* locus. (*A*) Schematic representation of *PiT1* alleles and targeting vector representing intronic DNA (horizontal lines), *PiT1* exons (black boxes) a *neo* cassette flanked by *frt* sites (unfilled box) and a diphtheria toxin cassette (hatched box). Heterozygous *PiT1^neo/+^* mice were produced as described. *Flp*-mediated recombination at *frt* sites generates the *PiT1^lox^* allele, whereas *Cre*-mediated recombination at *loxP* sites (open triangles) generates the *PiT1* ^*Δ5*^ allele. The positions of restriction sites used for Southern blot screening are shown: E, *EcoR*V; N, *Nhe*I; A, *ApaL*I. (*B*) Southern blot analysis of *EcoR*V-digested DNA from G418-resistant ES cell clones. The wild-type fragment is 33 kb in size, whereas correct recombination generates a 15.4 kb fragment using a 5′ probe and a 17.3 kb fragment using a 3′ probe (*bar*). A correctly targeted clone is represented (#18). (*C*) Genotype analysis of mice heterozygous for the various *PiT1* alleles. Southern blots were performed on *ApaL*I or *Nhe*I-digested kidney DNA isolated from mice as indicated, using 5′ and 3′ probes. (*D*) PCR genotyping of mice from tail genomic DNA. Primer positions (*triangles*) are indicated in (*A*) and their sequences are reported in Table S1. (*E*) Northern blot of E12.5 whole embryo total RNA as indicated, probed with ^32^P-labeled *PiT1* coding region cDNA. (*F*) Real-time RT-PCR analysis of the expression of the wild-type *PiT1* and *PiT2* alleles in E11.5 embryos from the *PiT1* allelic mutant series, as indicated. Note that *PiT2* is overexpressed in situation when the expression *PiT1* becomes very low. * and # indicate significant differences as compared to wild-type controls with *P*<0.05 and *P*<0.001, respectively (Student's t test).

To determine the *in vivo* function of PiT1, we targeted the *PiT1* locus by homologous recombination in ES cells and generated an allelic series of mutations ranging from mild impairment to elimination of PiT1 function. We inserted *loxP* sites 5′ and 3′ to *PiT1* exon 5 and a neomycin resistance (*neo*) cassette flanked by *frt* sites in intron 5 (Fig. 1A). Cre-mediated deletion of exon 5 is expected to produce a frame shift of the remaining sequence and convert the floxed *PiT1* allele to a null allele. Correctly targeted ES cells were identified by Southern blot analysis (Fig. 1B and C), chimeric mutant mice derived and *PiT1^neo/+^* heterozygous mice obtained (Fig. 1D).

*PiT1^neo/neo^* mice resulting from *PiT1^neo/+^* intercrosses were born at a lower than expected Mendelian frequency (Table S2). Analysis of *PiT1^neo^* transcripts demonstrated an aberrant splicing due to the presence of cryptic splice sites resulting in a hybrid mRNA containing an additional portion of *PiT1* intron 5 and the coding sequence of *neo* (Fig. S1A and B). Translation of the *PiT1^neo^* transcript would produce a non-functional PiT1 protein lacking exons 6 to 11 due to the presence of an in frame stop codon 65 bases downstream to exon 5. Accordingly, wild-type *PiT1* mRNA levels are significantly reduced both in *PiT1^neo/+^* and *PiT1^neo/neo^* E11.5 embryo (35% and 15% of the wild-type, respectively) (Fig. 1F). These results indicate that *PiT1^neo^* is a hypomorphic allele, leading to a reduction in the amount of functional *PiT1* gene expression, most probably accounting for the non-Mendelian frequency of *PiT1^neo/neo^* pups (Table S2). To excise the *neo* cassette *PiT1^neo/+^* animals were crossed to ACTB:FLPe mice [24], leading to *PiT1^lox/+^* offspring. A Mendelian distribution was observed in progeny obtained from *PiT1^lox/+^* intercrosses and no phenotypic difference was observed among genotypes, demonstrating that the *PiT1^lox^* allele is functionally equivalent to the wild-type allele. Accordingly, the level of wild-type *PiT1* mRNA was found normal in both *PiT1^lox/+^* and *PiT1^lox/lox^* mice (Fig. 1F).

To generate *PiT1*-null animals, *PiT1^neo/+^* mice were crossed with the *PGK-Cre* deleter strain mice [25]. The deletion was confirmed by Southern blot, PCR and northern blot analysis (Fig. 1C to E). *PiT1* ^Δ*5/+*^ mice appeared indistinguishable from *PiT1^+/+^* littermates, were viable and fertile producing offspring in normal Mendelian ratios when crossed with wild-type animals (Table 1). However, no *PiT1* ^Δ*5/*Δ*5*^ offspring were found among 208 newborn pups from heterozygote intercrosses, indicating that *PiT1* disruption results in embryonic lethality (Table 1).

**Table 1:** Genotypes of the progeny obtained from heterozygous *PiT* ^*Δ5/+*^ mice intercrosses.

Stage	Litters (n)	Embryos (n)	*PiT1^+/+^* a	*PiT1* ^*Δ5/+*^	*PiT1* ^*Δ5/Δ5*^ (alive)^b^	*PiT1* ^*Δ5/Δ5*^ (dead)
E3.5	3	27	7 (26%)	13 (48%)	7 (26%)	0
E9.5	3	26	7 (27%)	13 (50%)	6 (23%)	0
E10.5	6	52	13 (25%)	24 (46%)	15 (29%)	0
E11.5	15	127	34 (27%)	61 (48%)	32 (25%)	0
E12.5	19	162	41 (25%)	79 (49%)	37 (23%)	5 (3%)
E13.5	10	79	22 (28%)	43 (54%)	0**	14 (18%)
E14.5	4	38	11 (29%)	22 (58%)	0*	5 (13%)
E15.5	4	35	11 (31%)	24 (69%)	0*	0
Birth (P1)	25	208	60 (29%)	148 (71%)	0**	0
Total	89	754	206	427	97	24

*^a^*Absolute number and frequency (%).

*^b^*Surviving embryos were defined as those with beating hearts. No surviving *PiT1* ^*Δ5/Δ5*^ embryos were found alive past E12.5 stage.

*, ** significant differences between observed frequency of living *PiT1* ^*Δ5/Δ5*^ and expected frequency of 25%, according to Mendelian distribution of the genotypes with *P*<0.01 and *P*<0.001, respectively. Significance of distribution was calculated using χ^2^ test.

Similarly, *PiT1^neo/^* ^Δ*5*^ compound heterozygotes resulting from *PiT1^neo/+^* and *PiT1* ^Δ*5/+*^ matings were not viable (Table S3). Wild-type *PiT1* mRNA levels in E12.5 *PiT1^neo/^* ^Δ*5*^ embryos represented 6% of normal levels whereas *PiT2* expression was increased, as was seen in *PiT1^neo/neo^* and *PiT1* ^Δ*5/*Δ*5*^ mice (Fig. 1F). Hence, together with the *PiT1^+/+^*, *PiT1^neo/+^, PiT1* ^Δ*5/+*^ *, PiT1^neo/neo^* and *PiT1* ^Δ*5/*Δ*5*^, these mice contribute to the establishment of an allelic series of mutant mice in which *PiT1* is expressed from 100% to 0% (Fig. 1F).

### Mid-Gestation Lethality Due to Defective Liver Development and Anemia in *PiT1* ^*Δ5/Δ5*^ Embryos

**Figure 2:**
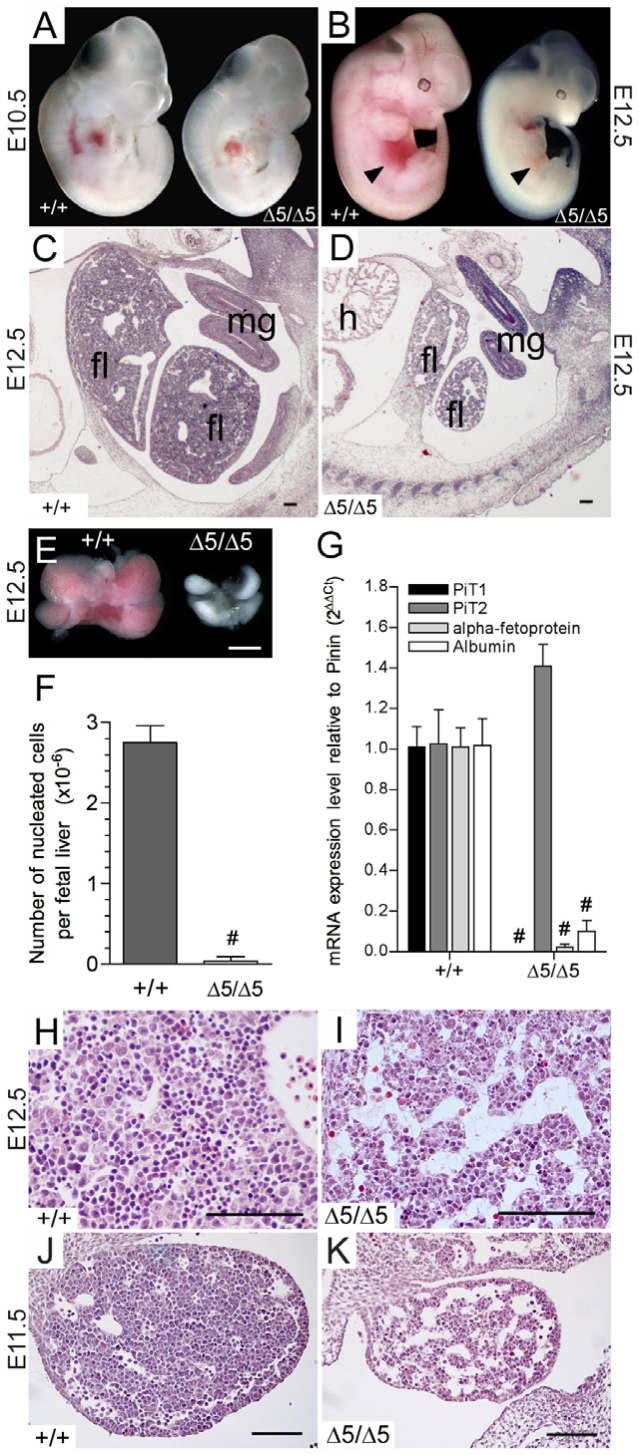
Disruption of the *PiT1* gene leads to defective liver development and anemia. (*A*–*B*) Comparison between wild-type and *PiT1* ^*Δ5/Δ5*^ embryos at E10.5 (*A*) and E12.5 (*B*). Note the reduced size, pale appearance and small liver (black arrow) of the E12.5 mutant embryos. (*C, D*) Haematoxilin and eosin (H&E) staining of E12.5 sections from *PiT1^+/+^* (*C*) and *PiT1* ^*Δ5/Δ5*^ embryos (*D*). fl: fetal liver; mg: mid-gut loop; h: heart. Bar, 100 µm. (*E*) Morphological appearance of wild-type and mutant E12.5 fetal livers. Bar, 0.5 mm (*F*) Fetal liver nucleated cell counts in E12.5 wild-type and mutant embryos. (*G*) Quantification of *PiT1*, *PiT2*, albumin and a-fetoprotein mRNA expression by real-time RT-PCR in E12.5 *PiT1^+/+^* and *PiT1* ^*Δ5/Δ5*^ embryos. (*H*–*K*) H&E staining of E12.5 (*H,I*) and E11.5 (*J,K*) sagital sections of *PiT1^+/+^* and *PiT1* ^*Δ5/Δ5*^ livers illustrating the hypocellularity of the mutant livers. Bar, 100 µm. # indicate significant differences as compared to wild-type controls with *P*<0.001 (Student's t test).

To identify at which developmental stage *PiT1*-null embryos die, genotyping of litters harvested from serial timed matings from E3.5 to birth was performed (Table 1). Results revealed that living *PiT1* ^Δ*5/*Δ*5*^ embryos were detected up to E12.5, and that no *PiT1* ^Δ*5/*Δ*5*^ embryos survived after this stage. Until E9.5 no macroscopic abnormalities were observed in *PiT1* ^Δ*5/*Δ*5*^ embryos by gross anatomical and histological examinations. At E10.5, embryos were found to be slightly growth retarded, without morphological abnormalities (Fig. 2A). At E12.5, *PiT1* ^Δ*5/*Δ*5*^ embryos could be easily recognized by their significant growth retardation and profound anemia (Fig. 2B). The most striking defect was observed in the developing liver, occupying only a small fraction of the abdominal areas (Fig. 2C and D). Morphological examination showed that E12.5 mutant livers, composed of four lobes, were extremely pale and severely reduced in size as compared to normal livers (Fig. 2E). Total liver nucleated cell counts represented only 2% of wild-type livers at this stage (Fig. 2F). Real-time RT-PCR analysis of RNA extracted from E12.5 *PiT1^+/+^* and *PiT1* ^Δ*5/*Δ*5*^ embryos demonstrated that the level of albumin and α-fetoprotein transcripts in mutant embryos was markedly reduced, further suggesting the decrease in hepatoblast number or function (Fig. 2G). Histological analysis of *PiT1* ^Δ*5/*Δ*5*^ E12.5 livers further confirmed the reduced cellularity and increased empty space, indicating less dense packing of cells in the *PiT1* ^Δ*5/*Δ*5*^ as compared to the wild-type livers (Fig. 2H and I). Hypocellularity and presence of large sinuses in the mutant livers were already visible at E11.5 (Fig. 2J and K).

### Absence of Placental, Yolk Sac and Vascular Defects in *PiT1* ^*Δ5/Δ5*^ Embryos

We next evaluated whether defects in placenta, yolk sac or vasculature could be responsible of the observed anemia seen in *PiT1* ^Δ*5/*Δ*5*^ embryos. Wild-type and mutant placentas appeared similar, except that mutant placentas were paler (Fig. S2A). The chorionic plate and labyrinthine trophoblast layer were well developed in *PiT1* ^Δ*5/*Δ*5*^ placentas, and no differences in vascularization were evidenced on histological sections (Fig. S2B to E). In addition, we did not detect differences in placental cell proliferation and apoptotic signals between mutant and control placentas (Fig. S2F to I). This suggests that the observed anemia is highly unlikely to be caused by defects in placental development and subsequent failure to establish the maternal-fetal interface for oxygen and nutrient exchange.

**Figure 3:**
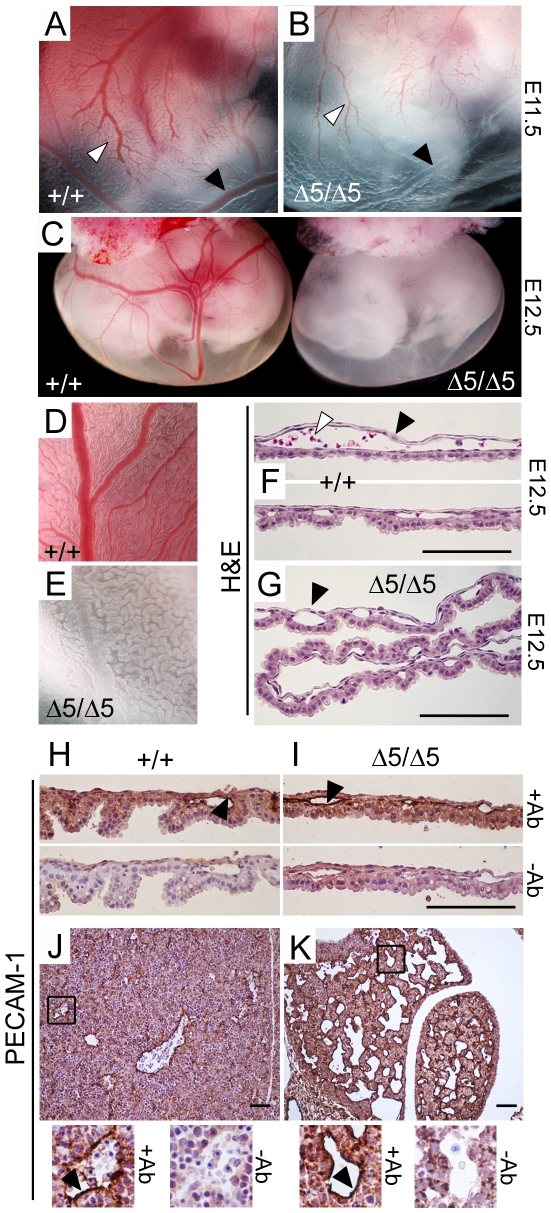
Absence of major vascular defects in *PiT1* ^*Δ*^ ^***5/Δ5***^ **mice.** (*A*–*B*) Yolk sac membranes from wild-type and PiT1^*Δ*5/*Δ*5^ E11.5 embryos illustrate the tree-like architecture of the vasculature (*white arrow*). Despite that mutant yolk sac appears white, vessels are present but often devoid of red blood cells (*black arrow*). (*C*) Gross appearance of wild-type (*left*) and PiT1^*Δ*5/*Δ*5^ (*right*) E12.5 embryos with intact yolk sac membranes. (*D*–*E*) Close examination of wild-type and mutant yolk sac shows empty vessels in the mutant yolk sac (*F*–*G*) H&E staining of yolk sac cross-sections from wild-type and *PiT1* ^*Δ5/Δ5*^ E12.5 embryos illustrate that, although null yolk sacs present with many vessels (*black arrow*), they are almost devoid of red blood cells (*white arrow*). (*H*–*I*) Anti-PECAM-1 IHC (*+Ab*) of *PiT1^+/+^* and *PiT1* ^*Δ5/Δ5*^ yolk sac cross-sections at E12.5 illustrating the presence of endothelial cells in both genotypes (*black arrow*). A negative control (*−Ab*) is shown to illustrate the specificity of the signal. (*J*–*K*) Anti-PECAM-1 IHC (*+Ab*) of *PiT1^+/+^* and *PiT1* ^*Δ5/Δ5*^ liver cross-sections at E12.5 illustrating the presence of endothelial cells in both genotypes. A negative control (*−Ab*) is shown to illustrate the specificity of the signal. Bar, 100 µm.

No hemorrhage was found in the mutant embryos, suggesting that a default in the vasculature is unlikely to be responsible for the anemia. At E11.5 the tree-like architecture of the vasculature was clearly visible (Fig. 3A and B), although severe decrease in red blood cells number impedes from visualizing the entire network. Close examination of E12.5 mutant yolk sac revealed a highly developed vasculature devoid of red blood cells (Fig. 3C to E). Histological examination and anti-PECAM-1 staining of endothelial cell of *PiT1* ^Δ*5/*Δ*5*^ yolk sac sections confirmed that major defects in the vasculature were absent from *PiT1* ^Δ*5/*Δ*5*^ mutants (Fig. 3F to I). Similarly, no difference in endothelial cell staining could be seen between wild-type and mutant sections throughout the entire embryo (data not shown). Anti-PECAM-1 staining on liver serial sections showed that although vessels appeared more numerous on the mutant livers, this was largely due to the severely reduced size of the liver (Fig. 3J and K). While these data do not exclude the possible existence of minor defects, they indicate that there were no major vascular defects shortly before the occurrence of embryonic death at E12.5.

### *PiT1* ^*Δ5/Δ5*^ Embryos Have Elevated Proportion of Circulating Primitive Erythrocytes and Identical Number of Hematopoietic Progenitors in the Liver

**Figure 4:**
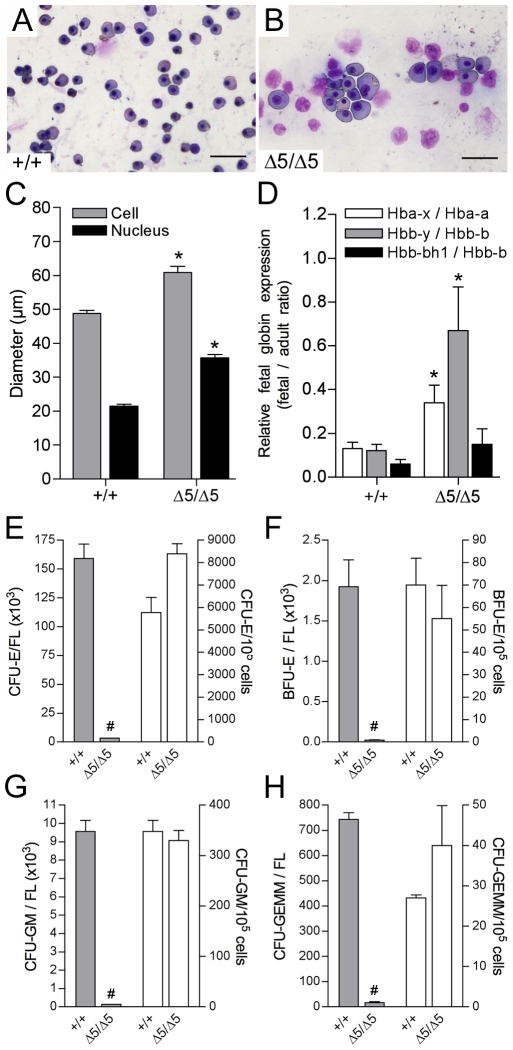
Elevated proportion of circulating primitive erythrocytes and identical number of hematopoietic progenitors per fetal liver in *PiT1* ^*Δ*^ ^***5/Δ5***^ **embryos.** (*A, B*) May-Grünwald/Giemsa staining of blood smears from E12.5 embryos. At E12.5 almost all blood cells are nucleated; enlarged cells represent primitive erythroid precursors. (*C*) Mean cell and nucleus diameter of erythroid cells in *PiT1^+/+^* and *PiT1* ^*Δ5/Δ5*^ E12.5 embryos, measured with the NIS-Elements AR 3.00 software. (*D*) RT-PCR analysis of the expression of globin chains in E12.5 *PiT1^+/+^* and *PiT1* ^*Δ5/Δ5*^ livers. Results are expressed as ratios between fetal and adult globin expression, as indicated. (*E*–*H*) *In vitro* differentiation of E12.5 fetal-liver cells from wild-type and *PiT1* ^*Δ5/Δ5*^ embryos. The number of CFU-E (*E*), BFU-E (*F*), CFU-GM (*G*), and CFU-GEMM (*H*) colonies per fetal liver (FL) or per 10^5^ nucleated fetal-liver cells are indicated. * and # indicate significant differences as compared to wild-type controls with *P*<0.05 and *P*<0.001, respectively (Student's t test).

Peripheral blood smears showed that the majority of erythroid cells in *PiT1* ^Δ*5/*Δ*5*^ mutants were larger than in the wild-type (Fig. 4A and B), which was confirmed by measuring the cell and nucleus mean diameters of erythroid cells in the *PiT1^+/+^* and *PiT1* ^Δ*5/*Δ*5*^ mice (Fig. 4C). Since primitive erythroid precursors are approximately twice as large and contain twice as much hemoglobin than their mature counterparts [33], this suggests that *PiT1* ^Δ*5/*Δ*5*^ embryos have a higher proportion of immature erythroid cells. We confirmed this by quantifying the expression of hemoglobin genes (Fig. 4D). Hemoglobin molecules contain globin chains derived both from the α-globin (Hbb-a) and β-globin (Hbb-b) gene loci. Although definitive erythroid cells in the mouse express α1-globin, α2-globin, β1-globin, and β2-globin, primitive erythroid cells in addition express ζ-globin (Hba-x), βH1-globin (Hbb-bh1), and εy-globin (Hbb-y) [33], [34]. We found that the amount of adult and fetal forms of globin chains in the mutant represented 2 to 9% and 19 to 25% of the wild-type, respectively. Accordingly, the ratios between fetal and adult forms of hemoglobin was higher in E12.5 *PiT1* ^Δ*5/*Δ*5*^ livers than in the wild-type (Fig. 4D), suggesting that the maturational globin switching may be impaired or delayed in *PiT1*-deficient embryos. Alternatively, a defect in the developmental niche provided by the fetal liver for the maturation and enucleation of primitive erythrocytes may explain this phenotype [35].

We conducted *in vitro* progenitor assays to characterize whether the erythropoietic defect in *PiT1* ^Δ*5/*Δ*5*^ embryos could result from either an intrinsic defect in hematopoietic progenitors or a failure in the ability of the fetal-liver microenvironment to support erythropoiesis. Single cell suspensions cultured in methylcellulose showed that the absolute number of CFU-E, BFU-E, CFU-GM and CFU-GEMM colonies per *PiT1* ^Δ*5/*Δ*5*^ fetal liver were reduced 48-, 90-, 74- and 46-fold, respectively, as compared with values in wild-type littermates (Fig. 4E to H). However, when results were expressed as number of colonies per 10^5^ nucleated fetal-liver cells, no significant difference was found between normal and mutant livers suggesting that a similar fraction of *PiT1* ^Δ*5/*Δ*5*^ and wild-type fetal livers are composed of hematopoietic cell progenitors (Fig. 4E to H). The ratio of BFU-Es to CFU-Es was similar in *PiT1^+/+^* and *PiT1* ^Δ*5/*Δ*5*^ embryos, suggesting that there was no defect in the terminal differentiation of erythroid progenitors. Both the size of CFU-E, BFU-E, CFU-GM and CFU-GEMM colonies and the number of cells per colony were similar (836±94 and 766±58 cells/colony for *PiT1^+/+^* and *PiT1* ^Δ*5/*Δ*5*^, respectively). These results suggest that although *PiT1* ^Δ*5/*Δ*5*^ fetal livers were severely hypoplastic, some hematopoietic stem cells do migrate there and have the potential to give rise to all erythropoietic lineages. Because the *PiT1* ^Δ*5/*Δ*5*^ hematopoietic committed progenitor cells were able to proliferate and differentiate normally when tested *in vitro*, this suggests that the anemia seen in E12.5 *PiT1* ^Δ*5/*Δ*5*^ embryos is unlikely to arise from a hematopoietic cell-autonomous defect.

### Increased Apoptosis and Reduced Growth Rate in *PiT1* ^*Δ5/Δ5*^ Livers

**Figure 5:**
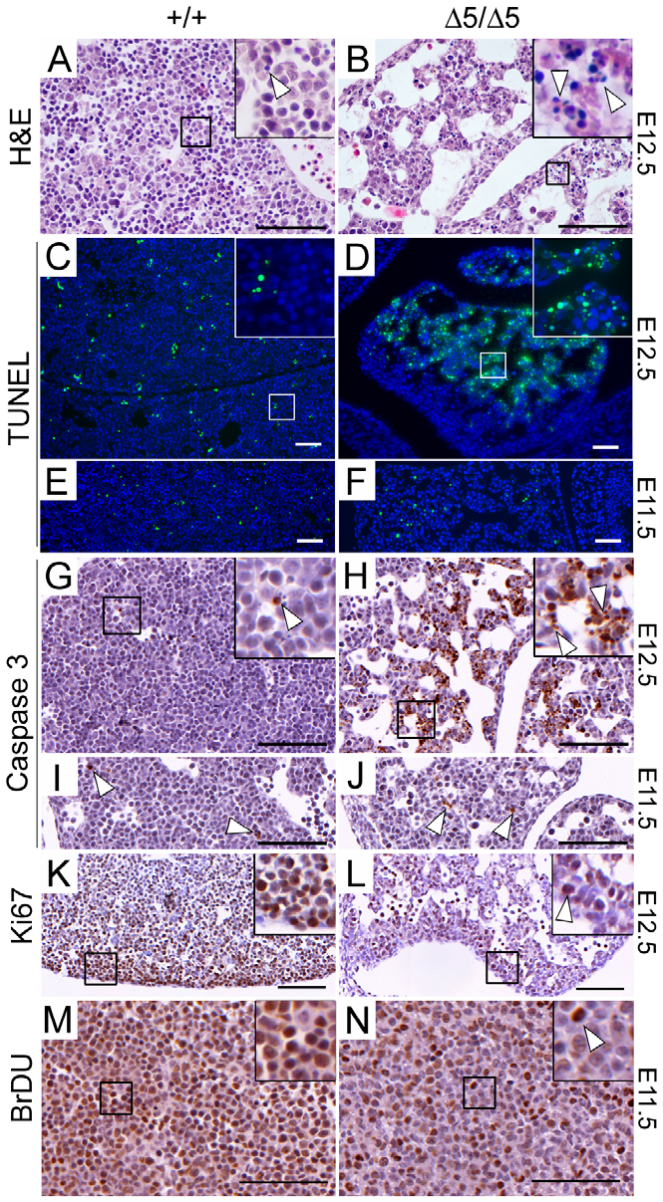
Apoptotic and proliferation defect in *PiT1*-deficient fetal livers. (*A*–*B*) H&E staining of E12.5 livers illustrating the presence of large sinuses in mutants. Higher magnification views (boxes) illustrate the abundance of pyknotic nuclei (*arrow*) in the mutant liver. (*C*–*D*) TUNEL analysis on sections from the same fetal livers shown in (*A*–*B*). Note the significant number of positively stained cells (*green*) in the *PiT1* ^*Δ5/Δ5*^ livers. Nuclei were stained with DAPI (*blue*). (*E*–*F*) TUNEL analysis on E11.5 fetal liver sections revealed that apoptotic signals were similar in wild-type and mutant livers. (*G*–*J*) Activated caspase 3 staining of E12.5 (*G,H*) and E11.5 (*I,J*), mutant liver sections. White arrows indicate examples of positively stained cells. (*K*–*L*) Ki67 staining shows that while overall cell density is reduced, proliferation is reduced but ongoing in mutant embryos (*arrow*). (*M*–*N*) Pulse BrDU labeling of E11.5 embryos. Positively labeled cells (arrow) are less numerous in the mutant. Bar, 100 µm.

**Figure 6:**
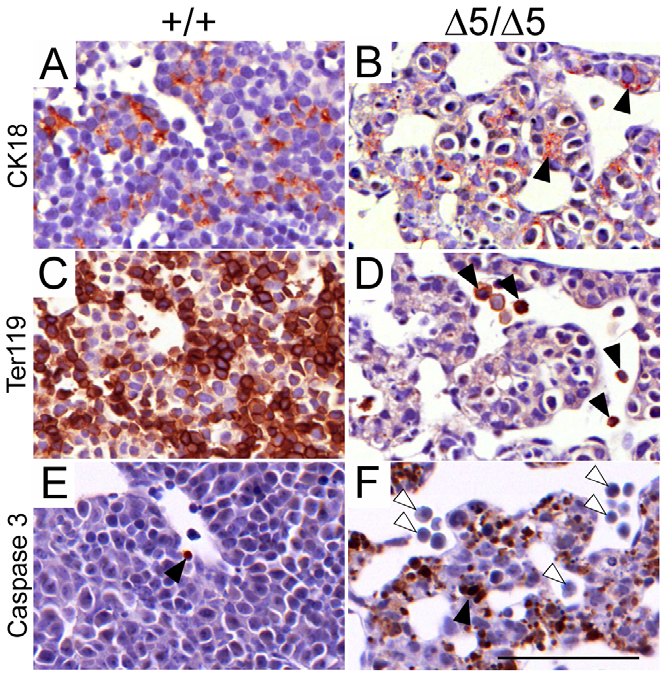
Apoptotic defects in PiT1 mutant fetal liver cells. (*A*–*B*) Cytokeratin 18 (CK18) staining (brown) reveals the presence of hepatoblasts in E12.5 mutant sections (*black arrows*). (*C*–*D*) Staining of Ter119 (brown) reveals great loss of erythroid cells in E12.5 mutant liver sections (*black arrows*). (*E*–*F*) Activated caspase 3 staining of E12.5 liver sections reveals excessive apoptotic activity in mutant liver sections (*black arrow*) that was not present in Ter119 positive cells (*white arrows*). Bar, 100 µm.

We next asked whether the reduction in liver cell density was caused by reduced proliferation or whether a loss of liver cells had occurred due to cell death. Histological sections revealed that most of the E12.5 mutant livers displayed abundant fragmented pyknotic nuclei, whereas only few were visible on control sections (Fig. 5A and B). TUNEL staining of liver sections confirmed the above observation (Fig. 5C to F). Of interest, the level of TUNEL staining in E11.5 mutant livers was comparable to that of wild-type controls (Fig. 5E and F). Immunodetection of activated caspase 3, revealed a significant number of apoptotic cells in E12.5 *PiT1* ^Δ*5/*Δ*5*^ livers, while few apoptotic cells were found in control livers (Fig. 5G and H). The level of activated caspase 3 in E11.5 mutant livers was comparable to that of wild-type (Fig. 5I and J). We next examined the proliferation status of PiT1-deficient livers. Staining with the proliferation marker Ki67 was less intense in sections of E12.5 *PiT1* ^Δ*5/*Δ*5*^ livers than in the wild-type (Fig. 5K and L). These results were confirmed by performing BrDU pulse-labeling of dividing cells in E11.5 pregnant females, and subsequent detection of BrDU-positive cells. Wild-type fetal livers showed heavy nuclear staining in the entire liver, whereas BrDU staining was reduced in *PiT1*-null samples directly demonstrating a subnormal growth rate in this organ (Fig. 5M and N). Antibodies recognizing the fetal hepatocyte marker cytokeratin 18 (CK18) revealed label-positive cells in PiT1 mutant embryos at E12.5, suggesting that, at least at this stage, developing hepatocytes were present (Fig. 6A and B). However, it was notable that CK18 staining revealed disorganized tissue architecture and that marker-positive cells were often rounded up or pyknotic. Staining against the erythroid cell marker Ter119 revealed a significant reduction in marker-positive cells in mutant livers (Fig. 6C and D), correlating with the loss of erythroid lineage cells. Activated caspase 3 staining of sections from the same livers revealed that Ter119-positive cells were not labeled in mutant sections whereas apoptosis was evident in CK18-expressing cells (Fig. 6E and F). Thus, while developing hepatocytes emerge in PiT1 mutant livers at E12.5, loss of hepatocytes by apoptosis is a frequent occurrence whereas the scarcity of Ter119-labeled cells in mutant livers is unlikely to correlate with apoptotic activity.

### *PiT1* Expression in the Liver

**Figure 7:**
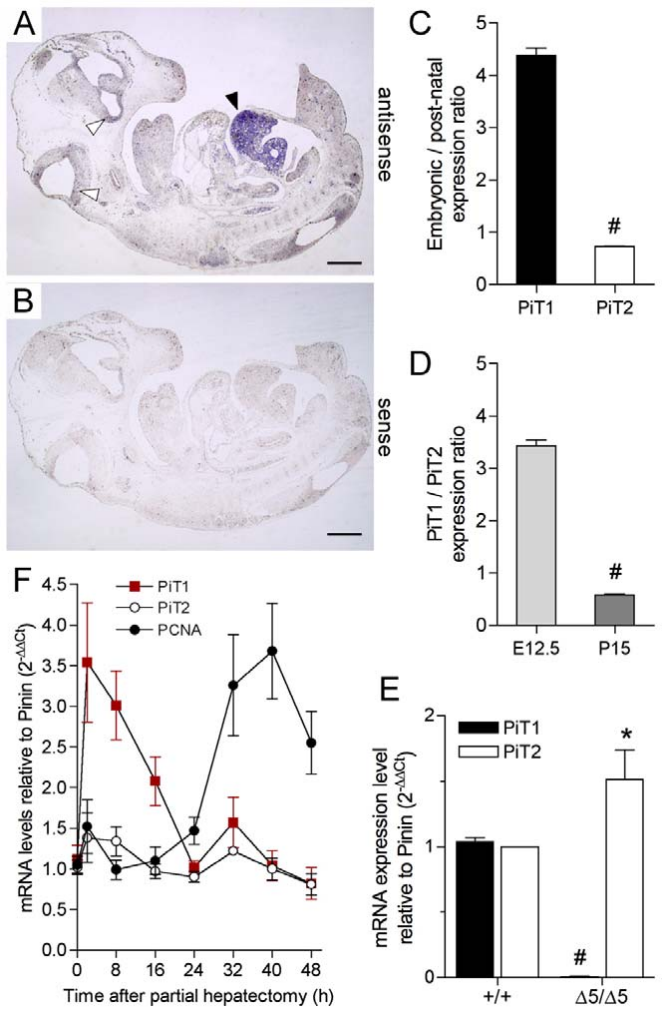
*PiT1* expression during liver growth. (*A*) *In situ* hybridization (ISH) of a wild-type E12.5 embryo shows heavy *PiT1* signal in the developing liver (black arrow), whereas low signal is detected in the brain (white arrows) and throughout the embryos. (*B*) ISH with the *PiT1* sense probe gives no signal. Bar, 1 mm. (*C*) Ratios of *PiT1* and *PiT2* mRNA expression levels between embryonic (E12.5) and post-natal (P15) wild-type livers, as determined by real-time RT-PCR. (*D*) *PiT1*/*PiT2* expression ratios in embryonic (E12.5) and post-natal (P15) livers, as determined by real-time RT-PCR. (*E*) Quantification of the expression of *PiT1* and *PiT2* in normal and *PiT1* ^*Δ5/Δ5*^ E12.5 livers by real-time RT-PCR. Note the 1.5-fold overexpression of *PiT2* in *PiT1*-null livers. (*F*) Gene induction after partial hepatectomy. Total cellular RNA collected in a time course after partial hepatectomy in wild-type mice was analyzed by real time RT-PCR for the expression of *PiT1*, *PiT2* and *PCNA* as a marker of proliferation. Results are reported after normalization to the expression of *Pinin*.

Although widespread, *PiT1* expression in adult mouse, rat and human is rather variable between tissues and is low in the adult liver [2], [7], [8]. Our *in situ* hybridization (ISH) experiments on E12.5 wild-type embryo sections demonstrated that *PiT1* was expressed at low levels throughout the embryo, except in the liver. Faint *PiT1* expression was detected in the developing brain as previously described [8], but this signal was much lower than the high level of expression found in the fetal liver (Fig. 7A and B). This result is in sharp contrast with the low level of expression of *PiT1* in adult livers, but is consistent with a role of *PiT1* during developmental liver growth. We confirmed these results by quantifying the expression of *PiT1* and *PiT2* in fetal E12.5 and post-natal P15 wild-type livers by real-time RT-PCR. The level of *PiT1* in the fetal liver was 4.4-fold higher than that in the post-natal liver, whereas fetal and post-natal *PiT2* expression were similar (Fig. 7C). Moreover, quantification of the relative expression of *PiT1* and *PiT2* in wild-type fetal and post-natal livers revealed that *PiT1* expression was 3.4-fold higher in fetal livers and 1.7-fold lower in post natal livers than *PiT2* (Fig. 7D), further illustrating the need for *PiT1* during developmental liver growth. Of importance, while *PiT1* was undetectable in *PiT1* ^Δ*5/*Δ*5*^ E12.5 livers, the expression of *PiT2* was 1.5-fold higher than in the wild-type livers (Fig. 7E), a value that is comparable to the increase found in the whole mutant embryo (Fig. 1F). To determine in which cell types PiT1 was most expressed, we performed immunostaining of PiT1 on wild-type and mutant sections using PiT1 antibodies from commercial or laboratory sources [9]. Unfortunately, we are unable to generate a specific signal using the available anti-mouse antibodies, and the expression of PiT1 in specific liver cell types during mouse development remains an open question. However, PiT1 has been localized in adult hepatocytes, but not in cholangiocytes, in rat livers [36]. Consistent with this finding, we further illustrated the role of *PiT1* in liver growth by performing partial hepatectomy on normal adult mice. In this model, the remnant liver undergoes rapid division to re-establish the original weight of the organ. Our results show that, although *PiT2* expression is unchanged, *PiT1* is highly (3.5-fold) induced within 2 h following partial hepatectomy (Fig. 7F) indicating that *PiT1* is likely to be essential during the early stage of compensatory liver growth. Although further experiments are necessary, this observation could be consistent with a higher expression of *PiT1* in developing versus adult fetal livers and the low proliferation rate of *PiT1* ^Δ*5/*Δ*5*^ livers.

### Proliferation of *PiT1* ^*Δ5/Δ5*^ Primary MEFs, but Not Na^+^-Pi Transport, Is Severely Impaired

**Figure 8:**
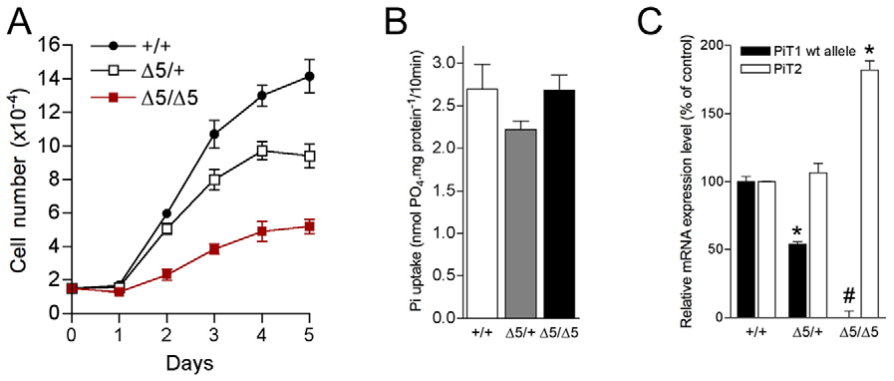
Disruption of *PiT1* in MEFs does not affect Na^+^-Pi cotransport but leads to reduced proliferation. (*A*) MEFs proliferation curve. *PiT1* ^*Δ5/Δ5*^ MEFs display a mean doubling time of 38 h, whereas *PiT1^+/+^* and *PiT1* ^*Δ5/+*^ MEFs had a growth rate of 18 h and 20 h, respectively. (*B*) Na^+^-Pi uptake in MEFs. Disruption of *PiT1* in MEFs does not modify the overall Pi uptake. (*C*) Quantification of the expression of *PiT1* and *PiT2* mRNAs in *PiT1^+/+^* and *PiT1* ^*Δ5/Δ5*^ MEFs by real-time RT-PCR. Note the 1.8-fold overexpression of *PiT2* mRNA in *PiT1*-null MEFs. * and # indicate significant differences as compared to wild-type controls with *P*<0.05 and *P*<0.001, respectively (Student's t test).

As genetic inactivation of *PiT1* resulted in embryonic lethality, together with decreased cell proliferation, we evaluated whether the absence of *PiT1* could also affect MEFs proliferation *in vitro*. Results show that *PiT1* ^Δ*5/*Δ*5*^ MEFs proliferated very slowly, displaying a mean doubling time of 38 h, a 2.1-fold increase compared with the doubling time of 18 h observed for *PiT1^+/+^* MEFs (Fig. 8A). *PiT1* ^Δ*5/+*^ MEFs had an intermediate growth rate (mean doubling time of 20 h). As the main reported function of PiT1 is to couple inward Pi uptake to the Na^+^ gradient across the cell plasma membrane, we characterized the Na^+^-dependent Pi uptake in *PiT1*-deficient MEFs. Interestingly, our results showed that Na^+^-Pi uptake in *PiT1*-null MEFs was unaffected (Fig. 8B), with no change in its kinetic properties (Vmax  = 19.2±0.7 and 19.1±1.7 nmol.mg prot^−1^ for *PiT1^+/+^* and *PiT1* ^Δ*5/*Δ*5*^ MEFs, respectively; Km  = 124.5±38.5 and 134.5±17.9 µM for *PiT1^+/+^* and *PiT1* ^Δ*5/*Δ*5*^ MEFs, respectively). While real-time RT-PCR confirmed that *PiT1* expression in *PiT1* ^Δ*5/*Δ*5*^ MEFs was abolished, this was associated with a 1.8-fold overexpression of *PiT2* mRNA (Fig. 8C), which could account for the maintenance of normal Na^+^-Pi transport in *PiT1-*null MEFs. Maintenance of a normal Pi uptake in *PiT1*-null MEFs supports the idea that the defect in MEF proliferation was not a consequence of Pi deprivation but resulted in a transport-independent function of PiT1, consistent with our recent data obtained in HepG2 and HeLa cells [9]. However, additional *in vivo* studies are necessary to assign the observed phenotype to a specific loss of either function of PiT1.

### Phenotype of Hypomorphic *PiT1^neo/neo^* Mice

Genotyping of litters from *PiT1^neo/+^* intercrosses showed that *PiT1^neo/neo^* pups were present at a lower frequency than expected (15% vs 25%) reaching only 5% at 2-weeks of age (Table S2), indicating that a low expression of *PiT1* is sufficient to bypass the embryonic lethality but results in partial postnatal lethality. The hypomorphic mice that survived were growth retarded (Fig. S3A to C). At birth, the mean weight of pups was 0.9±0.1 g for *PiT1^neo/neo^* and 1.5±0.2 g for wild-type or heterozygotes (Fig. S3A and B). At 2-3 weeks of age, the difference in weight was maximal (2-fold lower than controls). The weight of *PiT1^neo/neo^* adult mice ultimately remained approximately 1.3-fold lower than that of controls. The exact origin of the growth retardation is not known, but may be related to the anemia and subsequent hypo-oxygenation of growing tissues, suggested by the pale coloration of newborn hypomorphic pups (Fig. S3B). Peripheral blood analysis of 3-week-old mice showed mild but significant erythrocyte abnormalities in *PiT1^neo/neo^* mice as compared to litter-matched *PiT1^+/+^* mice (Table S4). The red blood cell counts, hematocrit, and hemoglobin concentration were significantly lower in hypomorphic than in wild-type mice. The mild anemia seen in the mutant was not explained by decreased formation of erythrocytes, since the reticulocyte number was 2.5-fold higher in the *PiT1^neo/neo^* mice (Table S4), consistent with an absence of abnormalities in bone marrow smears (data not shown). Together, these data are rather indicative of hemolytic anemia resulting from peripheral destruction of erythrocytes. Consistent with this possibility, *PiT1^neo/neo^* mice displayed a significant splenomegaly, whereas the size of the liver, brain and kidneys were normal (). Similarly, peripheral blood smears showed numerous abnormal red cell morphology, including echinocytes, spherocytes, and schizocytes (Fig. S3E). The origin of the observed phenotype is unknown and can arise either from decreased red cell membrane fluidity or defects in microvascular environment in the *PiT1^neo/neo^* mice. The absence of vessel defect in the *PiT1* ^Δ*5/*Δ*5*^ mice argues against the latter hypothesis, although detailed studies must be performed. Analysis of lipid membrane composition of mutant and control red blood cells demonstrated significant changes (Table S5). Monounsaturated fatty acid were increased, whereas polyunsatured fatty acid where decreased in *PiT1^neo/neo^* mice as compared to wild-type littermates: mutant red blood cells had higher levels of 16∶1n-7 and 18∶1n-9 and lower levels of 18∶0 fatty acids. However, such changes are not known to be associated with a change in membrane fluidity and may reflect the difference in the lipid membrane composition between erythrocytes and reticulocytes [37]. This latter possibility is further supported by the increase in stearoyl-CoA desaturase index in *PiT1^neo/neo^* mice. More importantly, cholesterol concentration and mean cholesterol/phospholipid molar ratio were not different between mutant and control mice (Table S5), arguing against a striking change in erythrocytes membrane fluidity. It remains to be determined, however, whether the decrease of PiT1 *per se* in the erythrocyte membrane of *PiT1^neo/neo^* mice can account for altered erythrocyte membrane morphology.

### Phenotype of *PiT1^neo/^* ^*Δ5*^ Compound Heterozygotes

The compound heterozygous *PiT1^neo/^* ^Δ*5*^ mice express functional *PiT1* levels that are intermediate between the *PiT1* ^Δ*5/*Δ*5*^ and *PiT1^neo/neo^* mice (Fig. 1F). Accordingly, several phenotypic features were found to lie in severity between the null and hypomorphic *PiT1* mice. Embryonic lethality of *PiT1^neo/^* ^Δ*5*^ mice was found to arise later in development than for *PiT1* ^Δ*5/*Δ*5*^ counterparts, occurring between E14.5 and E16.5 (Table S3). At E14.5, *PiT1^neo/^* ^Δ*5*^ embryos were severely anemic (Fig. S4A). At this stage mutant livers were hypoplastic, whereas E12.5 mutants and control livers showed comparable histology (Fig. S4B to E). Consistent with results obtained in *PiT1* ^Δ*5/*Δ*5*^ embryos, no hemorrhage was found in the *PiT1^neo/^* ^Δ*5*^ fetuses and no gross vascular defect could be detected by examination of yolk sacs (Fig. S4F and G). *PiT1^neo/^* ^Δ*5*^ circulating nucleated erythrocytes displayed morphologic features similar to those observed in controls (Fig. S4H and I) but were 6.9-fold more numerous than in controls (Fig. S4J). In addition, the level of embryonic hemoglobin was increased in the compound heterozygotes (Fig. S4K), suggesting that the relative increase in the number of nucleated erythrocytes in *PiT1^neo/^* ^Δ*5*^ is likely to result from the persistence of primitive yolk sac-derived erythrocytes secondary to a liver-defect in definitive erythropoiesis production.

### Impaired *PiT1* Expression in Mice Does Not Affect Early Skeleton Development

**Figure 9:**
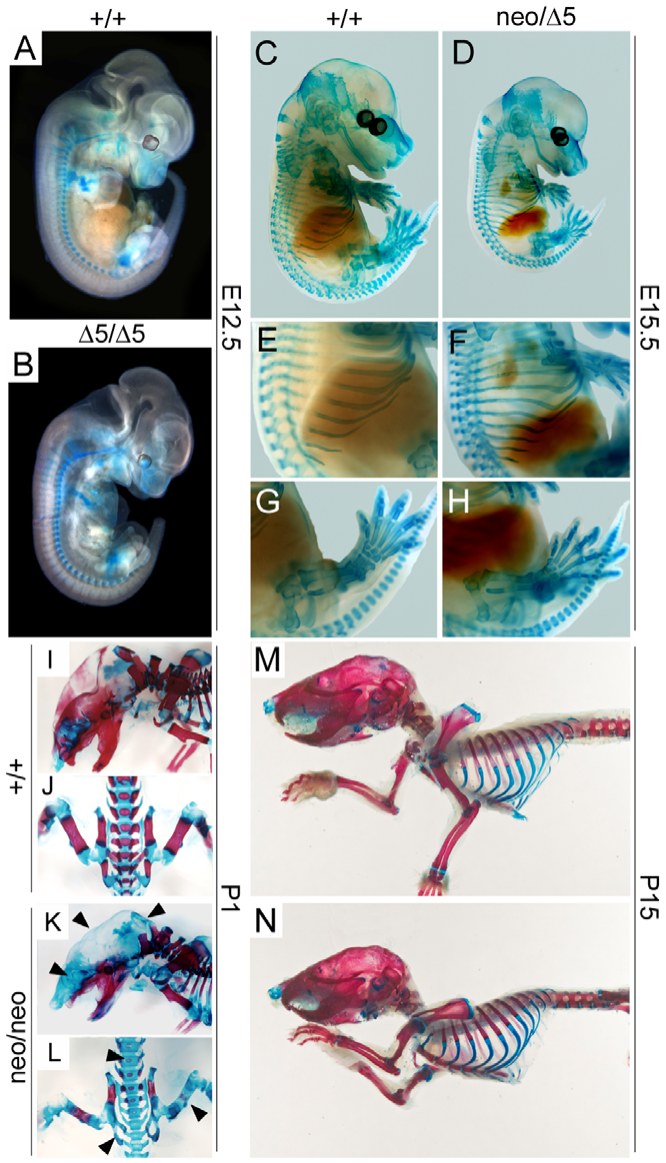
Impaired *PiT1* expression in mice does not affect early skeleton development. (*A*–*B*) Alcian blue staining of *PiT1^+/+^* and *PiT1* ^*Δ5/Δ5*^ E12.5 whole embryos. (*C*–*H*) Alcian blue staining of *PiT1^+/+^* and *PiT1^neo/^* ^*Δ5*^ E15.5 whole embryos. Higher magnification views (*E*–*H*) demonstrate no difference in skeletal development of the heterozygous compounds. (*I*–*L*) Alcian blue and alizarin red S double staining of one-day old *PiT1^+/+^* and *PiT1^neo/neo^* newborn pups. Note the lack of alizarin red staining in humerus, verterbraes, cranial vault and upper facial skeleton (black arrows). (*M*–*N*) Alcian blue and alizarin red S double staining of 15-day old *PiT1^+/+^* and *PiT1^neo/neo^* mice. No staining difference could be evidenced anymore.

Since PiT1 has been extensively studied in bone and cartilage, we investigated whether these tissues were affected in *PiT1* mutant mice. Alcian blue staining of the initial cartilage matrix revealed no difference between E12.5 *PiT1* ^Δ*5/*Δ*5*^ and *PiT1^+/+^* embryos (Fig. 9A and B), or later in development between E15.5 *PiT1^neo/^* ^Δ*5*^ and *PiT1^+/+^* embryos (Fig. 9C to H). Alcian blue/alizarin red S double staining of newborn *PiT1^neo/neo^* and wild-type skeletons showed an impaired mineralization of both long bones in which endochondral (arising from cartilaginous mold) ossification takes place, and in the cranial vault and portions of the upper facial skeleton that develop by intramembranous ossification without replacing a cartilaginous mold (Fig. 9I to L). However, these differences were not seen on all of the hypomorphic newborn pups (data not shown), suggesting that the impaired mineralization may have been already corrected. Consistent with this hypothesis, staining of 2-weeks-old skeletons demonstrated no detectable difference between *PiT1^neo/neo^* and *PiT1^+/+^* mice (Fig. 9M and N). Our data do not exclude an *in vivo* role for *PiT1* in bone and cartilage biology, but indicate that a normal expression of *PiT1* is not essential for early skeleton formation during mouse development.

## Discussion

In this report we have generated an allelic series of mutant mice in which *PiT1* is expressed from 100% to 0%. Disruption of *PiT1* leads to mid-gestation lethality due to severe defects in liver development, arising from reduced proliferation and massive apoptosis of liver cells. Impaired liver development causes dramatic anemia eventually leading to the death of the embryo. No placental, yolk sac or vascular defects were identified.

Considering the available data on *PiT1*, our findings are unexpected. *PiT1* is expressed in a wide variety of organs and cell types and, as a result, the general consensus is that this gene is ubiquitously expressed. However, this short-cut is misleading and although its expression is wide, there are large variations in *PiT1* expression levels from an organ to another, or under different physiological conditions [2], [7], [8]. Moreover, cell-type specific expression has not been addressed in most tissues except in bone, which showed that *PiT1* is expressed in a subset of hypertrophic chondrocytes and absent from osteoblasts during mouse development [20]. Our results clearly show that the embryo forms, develops and grows without major defects until E11.5 in the absence of *PiT1*, which demonstrates that *PiT1* is dispensable for early development. The over-expression of *PiT2* in *PiT1*-deficient embryos suggests that *PiT2* may compensate for the lack of *PiT1* up until this stage. However, decreased proliferation and increased apoptosis of *PiT1*-deficient fetal livers arise despite the over-expression of *PiT2* in the liver, demonstrating that PiT2 can not take over the function of PiT1 in the liver, and argues for distinct temporal or tissue-specific roles for PiT1 and PiT2. The novel mouse models described here will be useful tools for future studies addressing this question in more details.

Liver formation is a crucial checkpoint in fetal hematopoiesis and development. Livers from E11.5 and E12.5 *PiT1* ^Δ*5/*Δ*5*^ embryos were very small, contained hematopoietic precursors and disorganized islands of hepatocytes. A small liver can be the result of abnormal hematopoietic cell proliferation, as described in c-*myb*-deficient embryos [38], or abnormal erythroid cell proliferation and differentiation, as seen in *Rb^−/−^* embryos [39], [40]. However, we found normal *in vitro* proliferative and differentiative function of *PiT1* ^Δ*5/*Δ*5*^ hematopoietic committed progenitor cells making it unlikely that the anemia observed in *PiT1* ^Δ*5/*Δ*5*^ embryos can be attributed to a hematopoietic cell-autonomous defect. Moreover, although fetal livers are hypoplastic, erythroid progenitors have the ability to migrate there, arguing against a fetal liver homing defect. Liver cells from *PiT1* ^Δ*5/*Δ*5*^ embryos underwent a decrease in proliferation and a massive wave of apoptosis, suggesting that *PiT1* provides a proliferation and survival signal for hepatocytes during a defined stage of liver morphogenesis. This result was reinforced by the pattern of expression of *PiT1* in the embryo at mid-gestation showing a high and preferential expression in the liver, and the acute re-expression of *PiT1* following partial hepatectomy in adult liver, consistent with a crucial role of *PiT1* in liver growth. The molecular mechanisms by which *PiT1* mediates a pro-proliferative and anti-apoptotic function in the developing liver have yet to be clarified. In *PiT1*-null mice, the liver bud forms and the following proliferation period needed of expansion of the liver is highly affected. This phenotype is somewhat reminiscent of mice lacking RelA [41], Raf-1 [42], [43], MKK4 [44], ATF2 [45], MKK7 [46] or c-Jun [47]. There is no data in the literature linking *PiT1* to signaling pathways and our attempts to detect changes in the phosphorylation of relevant signaling pathways in the liver were unsuccessful (data not shown). As the mammalian target of rapamycin (mTOR) is a kinase that integrates signals from nutrients and growth factors to regulate cell growth and cell cycle progression coordinately, we also investigated the phosphorylation state of S6K1, S6, Akt1 and 4EBP1 proteins but could not show defects in *PiT1*-deficient MEFs (data not shown). We have shown that the proliferation rate of *PiT1*-null MEFs is decreased while the Na^+^-Pi transport activity remains unchanged, most probably due to the over-expression of *PiT2* that is observed in *PiT1*-deficient MEFs. This result is consistent with our recent data identifying a novel function of PiT1, not shared by PiT2, that is related to cell proliferation, and is independent of its Na^+^-Pi transport activity [9]. In *PiT1*-null fetal livers we show that *PiT2* is overexpressed but that this overexpression does not compensate for the loss of PiT1. Therefore it is tempting to speculate that as in MEFs, HepG2 and HeLa cells [9], a *PiT2* overexpression in mutant livers could compensate for a loss of Na^+^-Pi transport activity (as it is shared by PiT1 and PiT2), but not for a loss of a PiT1-specific function such as the proliferation-related function of PiT1. However, this remains an hypothesis since the generation of new animal models carrying mutations of either function of PiT1 (proliferation- or transport-related) is necessary to determine whether the observed phenotype of *PiT1*-null mice is due to a loss of either or both function of PiT1.

To date, most studies conducted on PiT1 have focused on the expression and Na^+^-Pi transport activity of PiT1 in tissues in which Pi biology plays an important role, mainly bone and vascular physiology. However, recent observations may be related to a transport-independent function of PiT1. Studies conducted in VSMCs have led to the proposal that an increase in Pi influx through the plasma membrane could lead to the to the transdifferentiation of VSMCs in osteoblast-like cells and calcification of the extracellular matrix under high extracellular Pi concentration [19]. Although PiT1 was irrefutably shown to be involved in this process, the mechanisms underlying the action of PiT1 is now being debated [48]. Notably, a recent report shows that under physiological conditions, the Pi-transport activity of PiT1 is saturated [49], implying that only an increase in the capacity (i.e. enhanced expression of functional Pi transporters) can mediate an increase in Pi influx. However, not only such an increase has not been evidenced but the expression of PiT1 in VSMCs is very low, making unlikely that PiT1 mediates its effect on vascular calficification through its Na^+^-Pi cotransport activity [48], [50]. While we were writing this paper, a technical report describing the generation of null and floxed alleles of *PiT1* has been published [51]. The authors have deleted exon 3 and 4 of *PiT1* which also led to embryonic lethality at mid-gestation around E12.5, although with a lower penetrance than in our model which may be due to differences in the genetic background used. Similarly to our results, they reported that *PiT1*-null embryos were anemic, although the origin of the anemia was not investigated and was based only on external observation. Of note, the authors reported possible defects in supercifial vasculature of yolk sac based on visual external observation [51]. However, although we also found that mutant yolk sacs appear white, we used specific endothelial cell immunostaining to show that there were no major vascular defects shortly before the occurrence of embryonic death at E12.5. In summary, although PiT1 does not seem to be essential to the development of a normal vasculature, it remains to be determined whether PiT1 is instrumental to a Pi-induced vascular calcification *in vivo* through the generation of animals carrying a tissue-specific deletion of PiT1.

The regulation of *PiT1* has been well documented in bone and cartilage models, due to the requirement of large amounts of Pi in bone physiology. *PiT1*, but not *PiT2*, mRNA and Na^+^-Pi transport were found to be regulated in osteoblast-like and chondrogenic cells by various factors, mainly Pi, IGF-1, BMP2 and TGF-β [10], [12], [13], [14], [16], [17]. Moreover, *PiT1*, but not *PiT2* is upregulated during osteoblast differentiation [15]. However, all these studies have been conducted *in vitro*, and it is still not clear whether the Na^+^-Pi transport function of PiT1 is implicated in these processes in normal physiological conditions. The weak expression of *PiT1* in bone, particularly in osteoblasts, its discrete expression in a subset of hypertrophic chondrocytes late in development [20], together with its low transport capacity [22] make it an unlikely candidate to face the tremendous Pi needs for bone and cartilage mineralization. The main role of *PiT1* in bone may rather be to mediate extracellular signals, as was recently proposed [52], independently, or additionally, of its transport function. Nevertheless, our data clearly show that there is no developmental defects in both vascular and cartilage compartments of *PiT1*-depleted mice. Although our results in hypomorphic *PiT1* mice show that, at birth, the mineralization of bone was delayed, its rapid normalization argues against a major predominant role for *PiT1* in skeleton mineralization. The availability of *PiT1^lox/lox^* mice will help at elucidating this particular point using appropriate Cre-expressing mice for generating bone and cartilage-specific deletion of *PiT1*.

In summary, through the establishment of a *PiT1* mutant allelic series we have uncovered a specific role of PiT1 in normal liver growth. We have shown that PiT1 is essential to embryonic development, and is non redundant as PiT2 could not compensate for the loss of PiT1. On the other hand, PiT1 seems not to be essential for skeleton and vasculature development. Although this study opens new avenues on the functions of the PiT protein family, the molecular mechanisms leading to the impairment of liver development remain to be elucidated, including open questions of whether the proliferation-associated function of PiT1 and/or the Na^+^Pi transport activity of PiT1 and PiT2 are involved in the observed phenotypes.

## Supporting Information

Figure S1 Aberrant splicing of the *PiT1^neo^* allele. (A) Schematic representation of the *PiT1^neo^* and *PiT1^Δ 5^* alleles, with black boxes representing *PiT1* coding sequences. The *neo* cassette and *loxP* sites are also depicted. Below the allele are diagrams representing the corresponding mRNAs produced by these alleles, as determined by the RT-PCR analysis shown in B using the primers indicated (arrow heads). (B) RNA isolated from the progeny of *PiT1^neo/+^* or *PiT1^Δ 5/+^* mice was reverse transcribed, and assayed by PCR using the primers indicated. Sequence analysis of these PCR products revealed the aberrant splicing of the *PiT1^neo^* allele, as illustrated in the diagram in A. The detection of similar size amplification products in samples derived from wild-type and *PiT1^neo/neo^* when primer pair F1R3 was used indicates that some wild-type *PiT1* mRNA is produced by the *PiT1^neo^* allele.
(0.31 MB TIF)

Figure S2 Absence of placental defect in *PiT1^Δ 5/Δ 5^* embryos. (A) External appearance of *PiT1^+/+^* (left) and *PiT1^Δ 5/Δ 5^* (right) placentas at E12.5. (B–C) Haematoxilin and eosin (H&E)-stained placental sections at E12.5. The size and histological appearance of the different layers are comparable between the wild-type and the mutant. Note the presence of less red blood cells in the mutant than in the wild-type. (D–E) Anti-PECAM-1 IHC of *PiT1^+/+^* and *PiT1^Δ 5/Δ 5^* placental sections at E12.5 illustrating the presence of endothelial cells in both genotypes. (F–G) Ki67 staining of placental sections from *PiT1^+/+^* and *PiT1^Δ 5/Δ 5^* mice. Most cells were positively labelled (white arrow), and few were negative (black arrow), demonstrating active and comparable cycling in both genotypes. (H–I) TUNEL analysis on placental sections from *PiT1^+/+^* and *PiT1^Δ 5/Δ 5^* mice demonstrate a low and comparable level of apoptotic cells (white arrow) in both genotypes. Sections were stained with DAPI to visualize the nuclei (blue). La: labyrinth; St: spongiotrophoblast; Gc: giant cells; Md: maternal decidua. Bars, 1 mm (A), 100 µm (B–I).
(7.39 MB TIF)

Figure S3 Phenotype of *PiT1^neo/neo^* mice. (A) Body weight as a function of age. Each point represents mean ± SD from 3 to 10 measurements. (B) Gross morphological appearance of *PiT1^+/+^* and *PiT1^neo/neo^* mice at birth. Note the smaller size and paler coloration of the mutant. (C) Gross appearance of *PiT1^+/+^* and *PiT1^neo/neo^* mice at 15-days of age. (D) Organ weight relative to body weight at 15-days of age. The spleen of the hypomorphic *PiT1* mouse is 2.4-fold larger than its wild-type counterpart. ** indicates significant differences as compared to wild-type controls with P<0.01 (Student's t test). (E) Peripheral blood smears from 3-weeks old *PiT1^+/+^* and *PiT1^neo/neo^* littermates. Numerous red cells with abnormal morphology are present, including echinocytes (black arrows), spherocytes (white arrows), and schizocytes (grey arrows).
(2.82 MB TIF)

Figure S4 Phenotype of *PiT1^neo/Δ 5^* compound heterozygotes. (A) Morphological appearance of *PiT1^neo/Δ 5^* E14.5 embryos (right), as compared to wild-type littermates (left). Note the severe anemia and slight decrease in size. (B–C) H&E staining of E14.5 sagital sections from *PiT1^+/+^* and *PiT1^neo/Δ 5^* livers. The mutant liver shows desorganized parenchyme, reduced cellularity and pyknotic nuclei (white arrow). (D–E) H&E staining of E12.5 sections from *PiT1^+/+^* and *PiT1^neo/Δ 5^* livers showing no difference between the two genotypes. Bar, 100 µm. (F–G) Close examination of yolk sac membranes from wild-type and *PiT1^neo/Δ 5^* E14.5 embryos demonstrate the presence of a highly developed vasculature (black arrows) characterized by a decrease in red blood cell number. (H–I) May-Grünwald/Giemsa staining of peripheral blood smears from E14.5 *PiT1^+/+^* and *PiT1^neo/Δ 5^* littermates. Note the similar morphology of red blood cells, but the higher percentage of nucleated erythrocytes (black arrows). (J) Quantification of the nucleated erythrocytes in the peripheral blood of E15.5 *PiT1^+/+^* and *PiT1^neo/Δ 5^* littermates. (K) RT-PCR analysis of the expression of globin chains in E15.5 *PiT1^+/+^* and *PiT1^neo/Δ 5^* littermate livers, and expressed as a ratio between fetal and adult expression. * and # indicate significant differences as compared to wild-type controls with P<0.05 and P<0.001, respectively (Student's t test).
(5.62 MB TIF)

Table S1 Primers used in this study.
(0.09 MB DOC)

Table S2 Genotypes of the progeny obtained by crossing heterozygous *PiT1^neo/+^* mice. ^a^ Surviving embryos were defined as those with beating hearts. *, *** significant differences between observed frequency of living *PiT1^neo/neo^* and expected frequency of 25%, according to Mendelian distribution of the genotypes, with P<0.05 and P<0.001, respectively. Significance of distribution was calculated using χ^2^ test.
(0.05 MB DOC)

Table S3 Genotypes of the progeny resulting from *PiT^neo/+^* and *PiT^Δ 5/+^*intercrosses. ^a^ Expected frequency according to Mendelian distribution of genotypes is 25% for each genotype. No surviving *PiT1^neo/Δ 5^* embryos were found alive past E16.5 stage. ^b^ Surviving embryos were defined as those with beating hearts. *, ** and *** significant differences between observed frequency of living *PiT1^neo/Δ 5^* and expected frequency of 25%, according to Mendelian distribution of the genotypes with P<0.05, P<0.01 and P<0.001, respectively. Significance of distribution was calculated using χ^2^ test.
(0.05 MB DOC)

Table S4 Hematologic variables in adult mice, according to genotype. Circulating blood of 3-week-old *PiT1^+/+^*, *PiT1^neo/+^* and *PiT1^neo/neo^* mice mice was analyzed on a Vet'ABC counter (SCIL, Viernheim, Germany). Reticulocyte count was determined on blood smears using a reticulocyte stain (Sigma). The data shown were obtained from a litter-matched group of mice (n = 3–4) and are representative of three experiments with independent groups of animals. Values are means ± SD. * and ** indicate significant differences between *PiT1^neo/neo^* and wild-type controls with P<0.05 and P<0.01, respectively (Student's t test). There was no significant difference between *PiT1^+/+^* and *PiT1^neo/+^* adults in any assay.
(0.06 MB DOC)

Table S5 Fatty acid, phospholipids and cholesterol profiles of *PiT1^+/+^*, *PiT1^neo/+^* and *PiT1^neo/neo^* erythrocyte membranes. ^a^ values are given as the mean from three mice in each group ± SD. The fatty acid contents are expressed as molar percentages. Percentages were significantly different from control by the two-tailed t-test with *P<0.05 and **P<0.01. ^b^ Unsaturation expressed as double bond index (calculated as the sum of each unsaturated fatty acid concentration multiplied by its double bond number and divided by the total unsaturated fatty acid concentration). SCD: stearoyl-CoA desaturase index (de novo synthesis of 18∶1n-9 from 18∶0); MUFA: monounsaturated; PUFA: polyunsaturated; EFA: essential. ^c^ values are given as the mean from three mice in each group ± SD. PE: phosphatidylethanolamine; PI: phosphatidylinositol; PS: phosphatidylserine; PC: phosphatidylcholine; SM: sphingomyelin; acidic: PI+PS; neutral: PE+PC+SM; PE+PS+PI: preferentially internal layer phospholipids; PC+SM: preferentially external layer phospholipids; PL: phospholipids.
(0.10 MB DOC)
