# Supplementary figures and images for: The Phosphate Transporter PiT1 (Slc20a1) Revealed As a New Essential Gene for Mouse Liver Development
Source: PLoS One. 2010 Feb 10;5(2):e9148. doi: 10.1371/journal.pone.0009148 (PMC2818845; doi:10.1371/journal.pone.0009148)

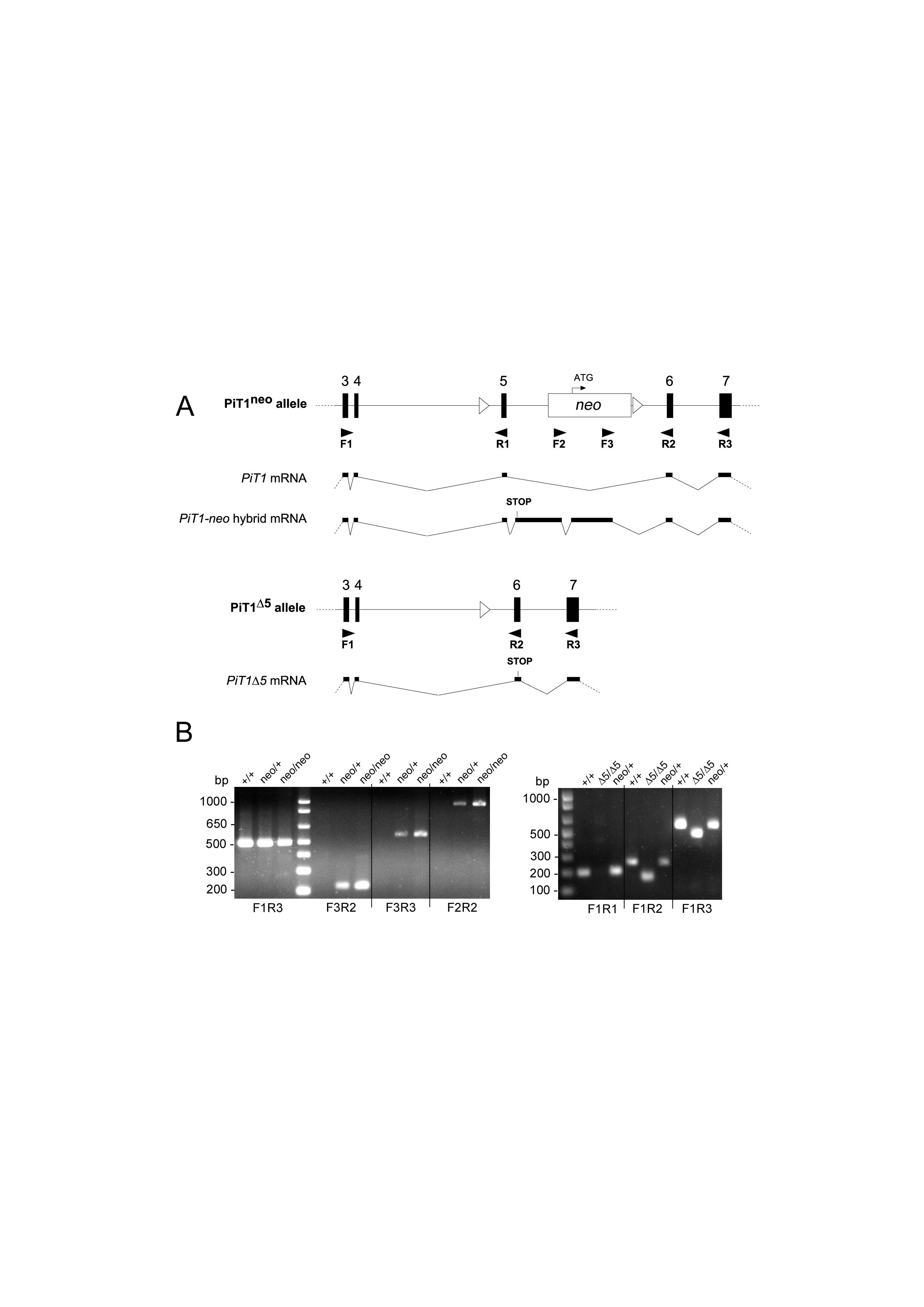

Supplement: Figure S1 — Aberrant splicing of the PiT1neo allele. (A) Schematic representation of the PiT1neo and PiT1Δ 5 alleles, with black boxes representing PiT1 coding sequences. The neo cassette and loxP sites are also depicted. Below the allele are diagrams representing the corresponding mRNAs produced by these alleles, as determined by the RT-PCR analysis shown in B using the primers indicated (arrow heads). (B) RNA isolated from the progeny of PiT1neo/+ or PiT1Δ 5/+ mice was reverse transcribed, and assayed by PCR using the primers indicated. Sequence analysis of these PCR products revealed the aberrant splicing of the PiT1neo allele, as illustrated in the diagram in A. The detection of similar size amplification products in samples derived from wild-type and PiT1neo/neo when primer pair F1R3 was used indicates that some wild-type PiT1 mRNA is produced by the PiT1neo allele. (0.31 MB TIF) [file pone.0009148.s001.tif]

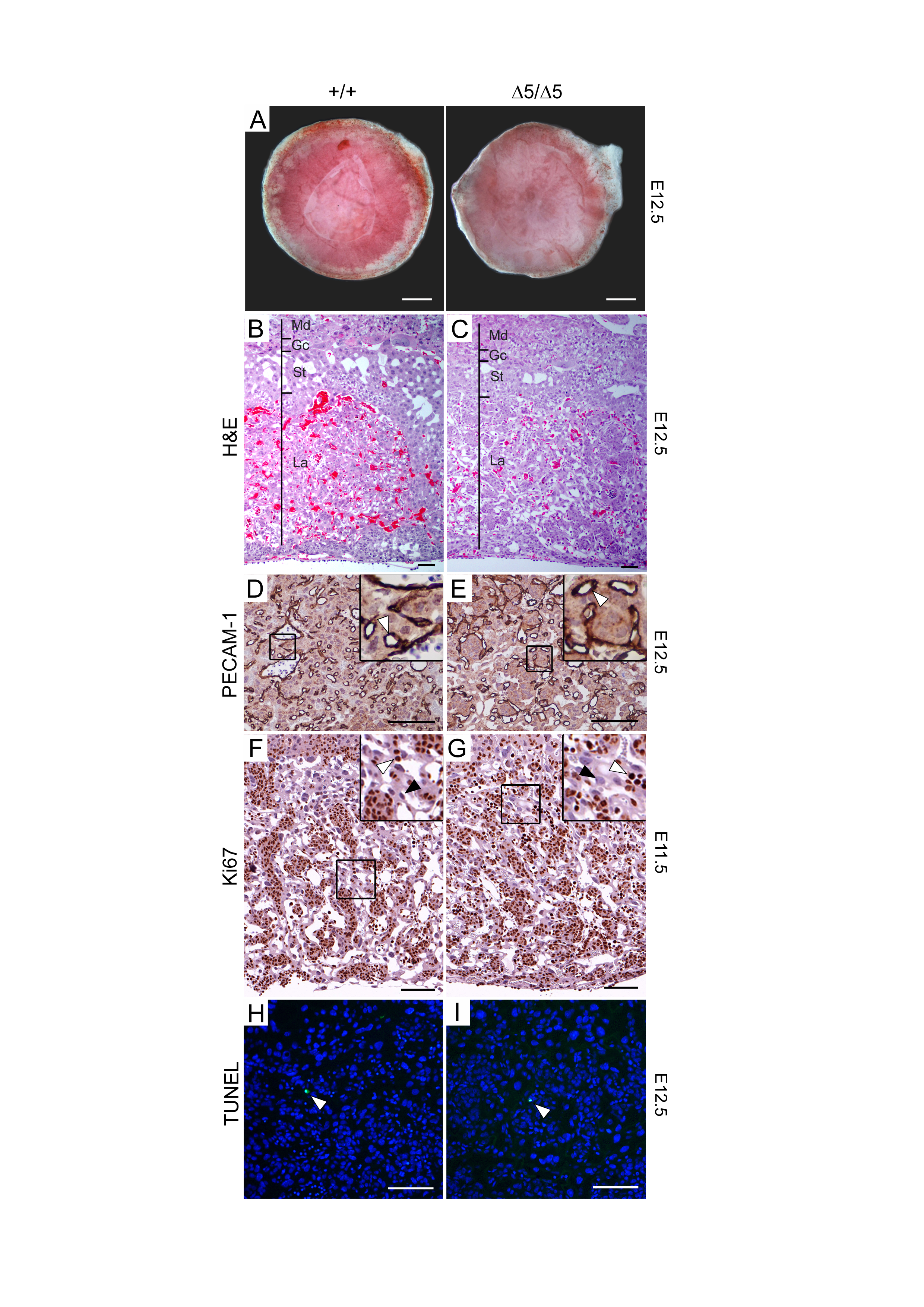

Supplement: Figure S2 — Absence of placental defect in PiT1Δ 5/Δ 5 embryos. (A) External appearance of PiT1+/+ (left) and PiT1Δ 5/Δ 5 (right) placentas at E12.5. (B–C) Haematoxilin and eosin (H&E)-stained placental sections at E12.5. The size and histological appearance of the different layers are comparable between the wild-type and the mutant. Note the presence of less red blood cells in the mutant than in the wild-type. (D–E) Anti-PECAM-1 IHC of PiT1+/+ and PiT1Δ 5/Δ 5 placental sections at E12.5 illustrating the presence of endothelial cells in both genotypes. (F–G) Ki67 staining of placental sections from PiT1+/+ and PiT1Δ 5/Δ 5 mice. Most cells were positively labelled (white arrow), and few were negative (black arrow), demonstrating active and comparable cycling in both genotypes. (H–I) TUNEL analysis on placental sections from PiT1+/+ and PiT1Δ 5/Δ 5 mice demonstrate a low and comparable level of apoptotic cells (white arrow) in both genotypes. Sections were stained with DAPI to visualize the nuclei (blue). La: labyrinth; St: spongiotrophoblast; Gc: giant cells; Md: maternal decidua. Bars, 1 mm (A), 100 µm (B–I). (7.39 MB TIF) [file pone.0009148.s002.tif]

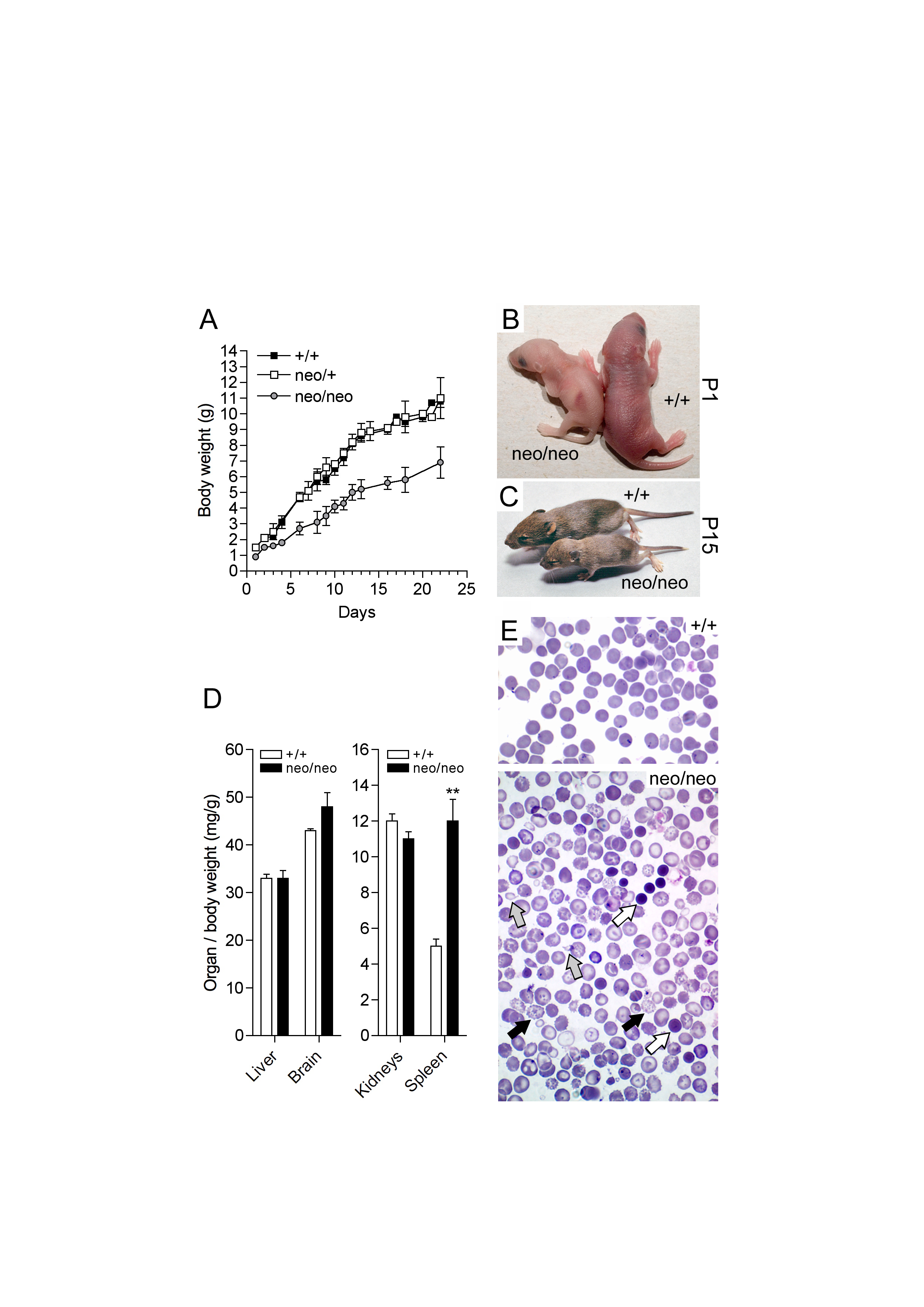

Supplement: Figure S3 — Phenotype of PiT1neo/neo mice. (A) Body weight as a function of age. Each point represents mean ± SD from 3 to 10 measurements. (B) Gross morphological appearance of PiT1+/+ and PiT1neo/neo mice at birth. Note the smaller size and paler coloration of the mutant. (C) Gross appearance of PiT1+/+ and PiT1neo/neo mice at 15-days of age. (D) Organ weight relative to body weight at 15-days of age. The spleen of the hypomorphic PiT1 mouse is 2.4-fold larger than its wild-type counterpart. ** indicates significant differences as compared to wild-type controls with P<0.01 (Student's t test). (E) Peripheral blood smears from 3-weeks old PiT1+/+ and PiT1neo/neo littermates. Numerous red cells with abnormal morphology are present, including echinocytes (black arrows), spherocytes (white arrows), and schizocytes (grey arrows). (2.82 MB TIF) [file pone.0009148.s003.tif]

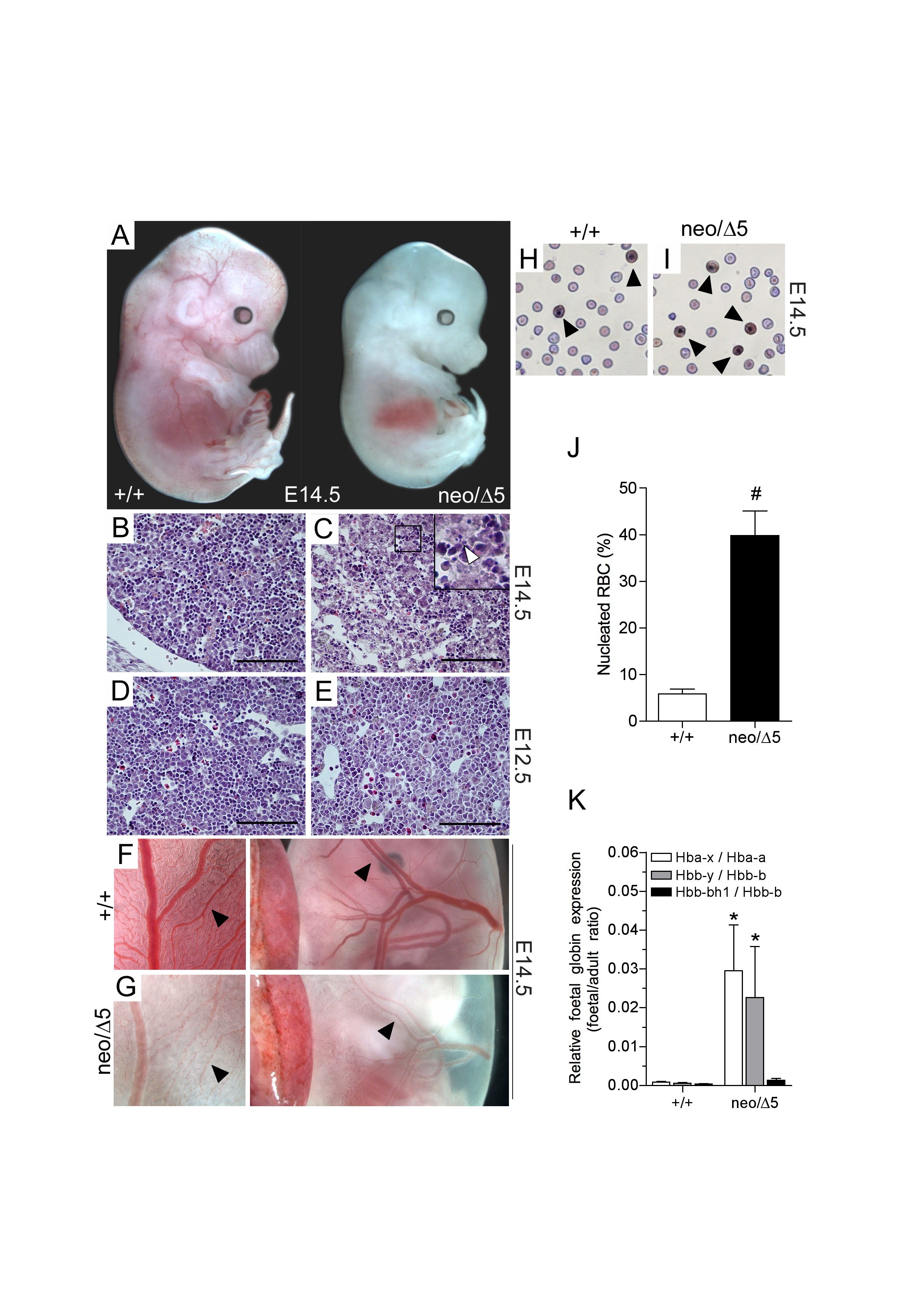

Supplement: Figure S4 — Phenotype of PiT1neo/Δ 5 compound heterozygotes. (A) Morphological appearance of PiT1neo/Δ 5 E14.5 embryos (right), as compared to wild-type littermates (left). Note the severe anemia and slight decrease in size. (B–C) H&E staining of E14.5 sagital sections from PiT1+/+ and PiT1neo/Δ 5 livers. The mutant liver shows desorganized parenchyme, reduced cellularity and pyknotic nuclei (white arrow). (D–E) H&E staining of E12.5 sections from PiT1+/+ and PiT1neo/Δ 5 livers showing no difference between the two genotypes. Bar, 100 µm. (F–G) Close examination of yolk sac membranes from wild-type and PiT1neo/Δ 5 E14.5 embryos demonstrate the presence of a highly developed vasculature (black arrows) characterized by a decrease in red blood cell number. (H–I) May-Grünwald/Giemsa staining of peripheral blood smears from E14.5 PiT1+/+ and PiT1neo/Δ 5 littermates. Note the similar morphology of red blood cells, but the higher percentage of nucleated erythrocytes (black arrows). (J) Quantification of the nucleated erythrocytes in the peripheral blood of E15.5 PiT1+/+ and PiT1neo/Δ 5 littermates. (K) RT-PCR analysis of the expression of globin chains in E15.5 PiT1+/+ and PiT1neo/Δ 5 littermate livers, and expressed as a ratio between fetal and adult expression. * and # indicate significant differences as compared to wild-type controls with P<0.05 and P<0.001, respectively (Student's t test). (5.62 MB TIF) [file pone.0009148.s004.tif]
