# Supplementary material for: The Phosphate Transporter PiT1 (Slc20a1) Revealed As a New Essential Gene for Mouse Liver Development
Source: PLoS One. 2010 Feb 10;5(2):e9148. doi: 10.1371/journal.pone.0009148 (PMC2818845; doi:10.1371/journal.pone.0009148)
Supplement: Table S1 — Primers used in this study. (0.09 MB DOC) [file pone.0009148.s005.doc]

**Table S1.** Primers used in this study

| Application | Gene | Sense (5’-3’) | Antisense (5’-3’) | Amplicon size | Ta (°C) | Cycles |
| --- | --- | --- | --- | --- | --- | --- |
| BAC subcloning (5') | PiT1 | ATACCGCGGCGCCGCTATTGTTATTTTCT | TGTCTAGAGGCTTGCTTCAGGGTCACTA | 508 | 55 | 30 |
| BAC subcloning (3') | PiT1 | GCTCTAGATGTTTGCACTTACCGTCAGG | TCGCCCGGGAGCAAATACCCAGTCAACCAA | 505 | 55 | 30 |
| 5' loxP site targeting (5') | PiT1 | GACTAGTTTGTACTTGCTATGCCTCTGT | GGAATTCGATATCTAAGTCCCCGTTGTCATAGAA | 235 | 55 | 30 |
| 5' loxP site targeting (3') | PiT1 | GCGGATCCGTATGGCCCCTGCTCTATTT | ATACTCGAGCCCAAAGTCCATCTGTAAACC | 278 | 55 | 30 |
| 3' loxP site targeting (5') | PiT1 | CGGGTAACTGAGAGATGGATAGATTGGAGC | GGAATTCGATATCTTGTTTTGTTCGGTGACTTACT | 337 | 55 | 30 |
| 3' loxP site targeting (3') | PiT1 | CGGGATCCAACAGCAGGGTATTTCTCACAT | GCTCTAGATATCTGCCGGTCTTTTCATTT | 293 | 55 | 30 |
| 5' genomic DNA probe | PiT1 | ATGTAGGGGACCAGGAAGCAATCT | TGTGCTGAGACTTGCCCTTGG | 682 | 55 | 30 |
| 3' genomic DNA probe | PiT1 | TTGATGCTACTGACAAGAAATG | CTAACAAGAACTCCTCCACAAA | 773 | 55 | 30 |
| Neo probe | PiT1 | GAGAGGCTATTCGGCTATGAC | CCCTCAGAAGAACTCGTCAAG | 750 |  |  |
| genotyping (primer #1 and #2) | PiT1 | CTCTTGCCTGTTTTTGTTTTTCC | AATGCTTATTTCCTGATGTCCTG | 300 (wt) / 404 (neo or lox) | 56 | 32 |
| genotyping (primer #1 and #3) | PiT1 | CTCTTGCCTGTTTTTGTTTTTCC | TGGTCCCATCCTGTGTTTCTT- | 256 (null) | 56 | 32 |
|  |  |  |  |  |  |  |
| Splicing (F1) | PiT1 | GGCAGAAGGGTGTCAAGTGGTC |  | N/A | N/A | N/A |
| Splicing (F2) | PiT1 | GAGAGGCTATTCGGCTATGAC |  | N/A | N/A | N/A |
| Splicing (F3) | PiT1 | CGGTGGGCTCTATGGCTTCTG |  | N/A | N/A | N/A |
| Splicing (R1) | PiT1 |  | AAGAGGTTGATTCCGATTGTGC | N/A | N/A | N/A |
| Splicing (R2) | PiT1 |  | TGGTACCCCACAGAGGAAGTTT | N/A | N/A | N/A |
| Splicing (R3) | PiT1 |  | CTATGCTGGTCTCCTCCTTCA | N/A | N/A | N/A |
|  |  |  |  |  |  |  |
| Northern/ISH | PiT1 | CTCCGTCAGTGCTATGTTTGG | CTATGCTGGTCTCCTCCTTCA | 715 | 56 | 35 |
| qPCR | PiT1 | CTTCCTTGTTCGTGCGTTCAT | AAGAGGTTGATTCCGATTGTGC | 102 | 60 | 40 |
| qPCR | PiT1 (wt allele) | CGTGCGTTCATCCTCCGTAAG | TCAAAGCCCAGCAACGGTG | 131 | 60 | 40 |
| qPCR | PiT2 | CCATCGGCTTCTCACTCGT | AAACCAGGAGGCGACAATCT | 80 | 60 | 40 |
| qPCR | Glucuronidase | TGGTATGAACGGGAAGCAATC | AATCCCATTCACCCACACAACT | 81 | 60 | 40 |
| qPCR | Pinin | ACCTGGAAGGGGCAGTCAGTA | ATCATCGTCTTCTGGGTCGCT | 86 | 60 | 40 |
| qPCR | Albumin | CGCCCATCGGTATAATGATTT | ATTTGGCATGCTCATCGTATG | 104 | 60 | 40 |
| qPCR | a-fetoprotein | CCAAAGCATTGCACGAAAA | TCCGGAACAAACTGGGTAAA | 118 | 60 | 40 |
| qPCR | PCNA | TGGAGCAACTTGGAATCCC | GTGGCTAAGGTCTCGGCATA | 92 | 60 | 40 |
| qPCR | Hbb-x | ATGTGGGAGAAGATGGCTGCT | CAGGTCGAAGTGGGGGAAGTA | 109 | 60 | 40 |
| qPCR | Hbb-a | AGCTGAAGCCCTGGAAAGGA | CTTCTTGCCGTGACCCTTGA | 110 | 60 | 40 |
| qPCR | Hbb-y | ACCTCCCAGACTTGCCATCA | CACAACAAGAAGCCTTCCCAA | 125 | 60 | 40 |
| qPCR | Hbb-bh1 | ACCTCACACCATGGTTCACTTC | AAACAATCAGGAGCCTTCCCA | 116 | 60 | 40 |
| qPCR | Hbb-b | CTGCCTTTAACGATGGCCTG | CATATTGCCCAGGAGCCTGA | 123 | 60 | 40 |
|  |  |  |  |  |  |  |
