# Supplementary material for: The Phosphate Transporter PiT1 (Slc20a1) Revealed As a New Essential Gene for Mouse Liver Development
Source: PLoS One. 2010 Feb 10;5(2):e9148. doi: 10.1371/journal.pone.0009148 (PMC2818845; doi:10.1371/journal.pone.0009148)
Supplement: Table S2 — Genotypes of the progeny obtained by crossing heterozygous PiT1neo/+ mice. a Surviving embryos were defined as those with beating hearts. *, *** significant differences between observed frequency of living PiT1neo/neo and expected frequency of 25%, according to Mendelian distribution of the genotypes, with P<0.05 and P<0.001, respectively. Significance of distribution was calculated using χ2 test. (0.05 MB DOC) [file pone.0009148.s006.doc]

**Table S2.** Genotypes of the progeny obtained by crossing heterozygous *PiT^neo/+^* mice

| Stage | Litters (n) | Observed frequency of living *PiT1^neo/neo^* (%) *^a^* |
| --- | --- | --- |
| E11.5 | 3 | 22 |
| E12.5 | 7 | 27 |
| E13.5 | 7 | 23 |
| E14.5 | 4 | 24 |
| E15.5 | 3 | 25 |
| E16.5 | 5 | 20 |
| E17.5 | 6 | 19 |
| P1 | 21 | 15* |
| P15 | 47 | 5*** |

*^a^* Surviving embryos were defined as those with beating hearts.

*, *** significant differences between observed frequency of living *PiT1^neo/neo^* and expected frequency of 25%, according to Mendelian distribution of the genotypes, with *P* < 0.05 and *P* < 0.001, respectively. Significance of distribution was calculated using χ^2^ test.
