# Supplementary material for: The Phosphate Transporter PiT1 (Slc20a1) Revealed As a New Essential Gene for Mouse Liver Development
Source: PLoS One. 2010 Feb 10;5(2):e9148. doi: 10.1371/journal.pone.0009148 (PMC2818845; doi:10.1371/journal.pone.0009148)
Supplement: Table S3 — Genotypes of the progeny resulting from PiTneo/+ and PiTΔ 5/+intercrosses. a Expected frequency according to Mendelian distribution of genotypes is 25% for each genotype. No surviving PiT1neo/Δ 5 embryos were found alive past E16.5 stage. b Surviving embryos were defined as those with beating hearts. *, ** and *** significant differences between observed frequency of living PiT1neo/Δ 5 and expected frequency of 25%, according to Mendelian distribution of the genotypes with P<0.05, P<0.01 and P<0.001, respectively. Significance of distribution was calculated using χ2 test. (0.05 MB DOC) [file pone.0009148.s007.doc]

**Table S3.** Genotypes of the progeny resulting from *PiT^neo/+^* and *PiT^∆5/+^* intercrosses

|  |  | Observed frequency (%) of each genotype ^a^ | | | | |
| --- | --- | --- | --- | --- | --- | --- |
| Stage | Embryos (n) | *PiT1^+/+^* | *PiT1^neo/+^* | *PiT1^∆5/+^* | *PiT1^neo/∆5^*  (alive) *^b^* | *PiT1^neo/∆5^*  (dead) |
| E11.5 | 24 | 6 (25%) | 5 (21%) | 7 (29%) | 6 (25%) |  |
| E12.5 | 71 | 18 (25%) | 17 (24%) | 19 (27%) | 17 (24%) | 0 |
| E13.5 | 37 | 10 (27%) | 10 (27%) | 8 (22%) | 9 (24%) | 0 |
| E14.5 | 91 | 23 (25%) | 25 (27%) | 22 (24%) | 20 (22%) | 1 (1%) |
| E15.5 | 73 | 18 (25%) | 19 (26%) | 17 (23%) | 15 (21%) | 4 (5%) |
| E16.5 | 96 | 25 (26%) | 24 (25%) | 22 (23%) | 6 (6%)* | 19 (20%) |
| E17.5 | 50 | 14 (28%) | 12 (24%) | 14 (28%) | 0** | 10 (20%) |
| Birth (P1) | 67 | 23 (34%) | 21 (31%) | 23 (34%) | 0*** | 0 |

*^a^* Expected frequency according to Mendelian distribution of genotypes is 25% for each genotype. No surviving *PiT1^neo/∆5^* embryos were found alive past E16.5 stage.

*^b^* Surviving embryos were defined as those with beating hearts.

*, ** and *** significant differences between observed frequency of living *PiT1^neo/∆5^* and expected frequency of 25%, according to Mendelian distribution of the genotypes with *P < 0.05*, *P < 0.01* and *P < 0.001*, respectively. Significance of distribution was calculated using χ^2^ test.
