# Supplementary material for: The Phosphate Transporter PiT1 (Slc20a1) Revealed As a New Essential Gene for Mouse Liver Development
Source: PLoS One. 2010 Feb 10;5(2):e9148. doi: 10.1371/journal.pone.0009148 (PMC2818845; doi:10.1371/journal.pone.0009148)
Supplement: Table S4 — Hematologic variables in adult mice, according to genotype. Circulating blood of 3-week-old PiT1+/+, PiT1neo/+ and PiT1neo/neo mice mice was analyzed on a Vet'ABC counter (SCIL, Viernheim, Germany). Reticulocyte count was determined on blood smears using a reticulocyte stain (Sigma). The data shown were obtained from a litter-matched group of mice (n = 3–4) and are representative of three experiments with independent groups of animals. Values are means ± SD. * and ** indicate significant differences between PiT1neo/neo and wild-type controls with P<0.05 and P<0.01, respectively (Student's t test). There was no significant difference between PiT1+/+ and PiT1neo/+ adults in any assay. (0.06 MB DOC) [file pone.0009148.s008.doc]

**Table S4**. Hematologic variables in adult mice, according to genotype

|  | *PiT1^+/+^* | *PiT1^neo/+^* | *PiT1^neo/neo^* |
| --- | --- | --- | --- |
| Red blood cells (10^6^/mm^3^) | 11.1 ± 0.5 | 11.3 ± 0.6 | 8.7 ± 1.2** |
| Hemoglobin (g/dl) | 18.1 ± 1.2 | 18.2 ± 1.2 | 14.6 ± 1.8** |
| Hematocrit (%) | 62.1 ± 3.6 | 65.2 ± 2.2 | 51.8 ± 5.4* |
| Mean corpuscular volume (µm^3^) | 56.2 ± 1.3 | 57.8 ± 1.3 | 59.8 ± 2.4* |
| Mean corpuscular hemoglobin (pg) | 16.4 ± 0.6 | 16.1 ± 0.3 | 16.8 ± 0.3 |
| Mean corpuscular hemoglobin concentration (g/dl) | 29.2 ± 1.1 | 27.9 ± 1.0 | 28.1 ± 0.6 |
| Red cell distribution width (%) | 12.8 ± 0.9 | 12.4 ± 0.9 | 16.0 ± 1.0* |
| Reticulocytes (%) | 5.2 ± 0.8 | 6.1 ± 1.1 | 13.2 ± 1.4** |
| White cells (10^3^/mm^3^) | 9.3 ± 1.8 | 8.8 ± 1.1 | 10.2 ± 5.2 |
| Lymphocytes (%) | 82.3 ± 4.4 | 86.8 ± 2.8 | 81.3 ± 1.2 |
| Monocytes (%) | 4.0 ± 0.4 | 3.5 ± 0.7 | 4.8 ± 0.6 |
| Granulocytes (%) | 13.7 ± 4.1 | 9.7 ± 2.3 | 13.9 ± 0.9 |
| Platelets (10^3^/mm^3^) | 676 ± 271 | 582 ± 332 | 814 ± 462 |
| Mean Platelet Volume (µm^3^) | 5. 0 ± 0.6 | 4.9 ± 0.4 | 5.6 ± 1.2 |

Circulating blood of 3-week-old *PiT1^+/+^* , *PiT1^neo/+^* and *PiT1^neo/neo^* mice mice was analyzed on a Vet’ABC counter (SCIL, Viernheim, Germany). Reticulocyte count was determined on blood smears using a reticulocyte stain (Sigma). The data shown were obtained from a litter-matched group of mice (*n* = 3-4) and are representative of three experiments with independent groups of animals. Values are means ± SD. * and ** indicate significant differences between *PiT1^neo/neo^* and wild-type controls with *P* < 0.05 and *P* < 0.01, respectively (Student’s t test). There was no significant difference between *PiT1^+/+^* and *PiT1^neo/+^* adults in any assay.
