# Supplementary material for: The Phosphate Transporter PiT1 (Slc20a1) Revealed As a New Essential Gene for Mouse Liver Development
Source: PLoS One. 2010 Feb 10;5(2):e9148. doi: 10.1371/journal.pone.0009148 (PMC2818845; doi:10.1371/journal.pone.0009148)
Supplement: Table S5 — Fatty acid, phospholipids and cholesterol profiles of PiT1+/+, PiT1neo/+ and PiT1neo/neo erythrocyte membranes. a values are given as the mean from three mice in each group ± SD. The fatty acid contents are expressed as molar percentages. Percentages were significantly different from control by the two-tailed t-test with *P<0.05 and **P<0.01. b Unsaturation expressed as double bond index (calculated as the sum of each unsaturated fatty acid concentration multiplied by its double bond number and divided by the total unsaturated fatty acid concentration). SCD: stearoyl-CoA desaturase index (de novo synthesis of 18∶1n-9 from 18∶0); MUFA: monounsaturated; PUFA: polyunsaturated; EFA: essential. c values are given as the mean from three mice in each group ± SD. PE: phosphatidylethanolamine; PI: phosphatidylinositol; PS: phosphatidylserine; PC: phosphatidylcholine; SM: sphingomyelin; acidic: PI+PS; neutral: PE+PC+SM; PE+PS+PI: preferentially internal layer phospholipids; PC+SM: preferentially external layer phospholipids; PL: phospholipids. (0.10 MB DOC) [file pone.0009148.s009.doc]

**Table S5.** Fatty acid, phospholipids and cholesterol profiles of *PiT1^+/+^*, *PiT1^neo/+^* and *PiT1^neo/neo^* erythrocyte membranes

|  |  | *PiT1^+/+^* | *PiT1^neo/+^* | *PiT1^neo/neo^* |
| --- | --- | --- | --- | --- |
| Fatty acids*^a^* | 16:0 | 38.1 ± 1.6 | 39.8 ± 1.1 | 43.3 ± 0.9* |
|  | 16:1n-7 | 0.3 ± 0.5 | 1.2 ± 0.7 | 1.7 ± 0.5* |
|  | 18:0 | 10.6 ± 0.6 | 8.7 ± 0.8 | 7.2 ± 0.7* |
|  | 18:1n-9 | 13.7 ± 0.3 | 14.5 ± 0.3 | 15.3 ± 0.6* |
|  | 18:2n-6 | 11.8 ±0.4 | 11.3 ± 0.6 | 12.2 ± 0.7 |
|  | 18:3n-3 | 0.3 ± 0.0 | 0.3 ± 0.2 | 0.4 ± 0.3 |
|  | 20:3n-6 | 1.3 ± 0.1 | 1.6 ± 0.1 | 1.4 ± 0.0 |
|  | 20:4n-6 | 15.9 ± 1.1 | 14.6 ± 0.0 | 11.8 ± 0.6* |
|  | 20:5n-3 | 0.2 ± 0.0 | 0.2 ± 0.1 | 0.2 ± 0.0 |
|  | 22:4n-6 | 2.4 ± 0.3 | 2.5 ± 0.1 | 2.0 ± 0.1 |
|  | 22:5n-6 | 0.9 ± 0.2 | 0.9 ± 0.1 | 0.6 ± 0.1* |
|  | 22:5n-3 | 1.0 ± 0.2 | 0.9 ± 0.1 | 0.7 ± 0.1 |
|  | 22:6n-3 | 3.6 ± 0.6 | 3.5 ± 0.0 | 3.1 ± 0.2 |
|  |  |  |  |  |
|  | MUFA | 13.9 ± 0.8 | 15.7 ± 0.4 | 17.0 ± 0.8** |
|  | PUFA | 25.2 ± 2.1 | 24.1 ± 0.2 | 19.8 ± 0.9** |
|  | EFA | 12.1 ± 0.4 | 11.6 ± 0.4 | 12.7 ± 0.4 |
|  | Unsaturation*^b^* | 147.3 ± 8.1 | 143.2 ± 1.1 | 128.4 ± 3.3* |
|  | n-6 | 32.3 ± 1.2 | 30.9 ± 0.5 | 28.0 ± 1.0* |
|  | n-3 | 5.0 ± 0.6 | 4.9 ± 0.4 | 4.5 ± 0.3 |
|  | n-6/n-3 | 6.4 ± 0.5 | 6.3 ± 0.6 | 6.3 ± 0.4 |
|  | SCD | 1.3 ± 0.1 | 1.7 ± 0.2 | 2.1 ± 0.3** |
|  |  |  |  |  |
| Phospholipids*^c^* | %PE | 49.7 ± 8.3 | 38.9 ± 6.0 | 38.9 ± 2.8 |
|  | %PI | 1.2 ± 2.0 | 2.3 ± 3.3 | 5.7 ± 5.1 |
|  | %PS | 19.2 ± 6.1 | 17.5 ± 2.0 | 19.4 ± 5.6 |
|  | %PC | 12.6 ± 3.6 | 19.8 ± 6.2 | 19.8 ± 3.7 |
|  | %SM | 17.4 ± 4.7 | 21.5 ± 1.2 | 16.2 ± 0.4 |
|  | PC/SM | 0.7 ± 0.1 | 0.9 ± 0.2 | 1.2 ± 0.2** |
|  | %acidic | 20.3 ± 4.6 | 19.8 ± 1.3 | 25.1 ± 1.0 |
|  | %neutral | 79.7 ± 4.6 | 80.2 ± 1.3 | 74.9 ± 1.0 |
|  | PE+PS+PI | 70.0 ± 8.2 | 58.7 ± 7.4 | 64.0 ± 3.9 |
|  | PC+SM | 30.0 ± 8.2 | 41.3 ± 7.4 | 36.0 ± 3.9 |
|  |  |  |  |  |
| Cholesterol | Chol. (µg/million) | 0.05 ± 0.01 | 0.04 ± 0.01 | 0.07 ± 0.02 |
|  | Chol. (µg/µl suspension) | 0.75 ± 0.15 | 0.56 ± 0,24 | 0.75 ± 0.24 |
|  | PL/CHOL | 3.0 ± 0.1 | 2.4 ± 0.2 | 3.2 ± 0.3 |
|  |  |  |  |  |

*^a^* values are given as the mean from three mice in each group ± SD. The fatty acid contents are expressed as molar percentages. Percentages were significantly different from control by the two-tailed t-test with *P<0.05 and **P<0.01.

*^b^* Unsaturation expressed as double bond index (calculated as the sum of each unsaturated fatty acid concentration multiplied by its double bond number and divided by the total unsaturated fatty acid concentration). SCD: stearoyl-CoA desaturase index (*de novo* synthesis of 18:1n-9 from 18:0); MUFA: monounsaturated; PUFA: polyunsaturated; EFA: essential.

*^c^* values are given as the mean from three mice in each group ± SD. PE: phosphatidylethanolamine; PI: phosphatidylinositol; PS: phosphatidylserine; PC: phosphatidylcholine; SM: sphingomyelin; acidic: PI+PS; neutral: PE+PC+SM; PE+PS+PI: preferentially internal layer phospholipids; PC+SM: preferentially external layer phospholipids; PL : phospholipids.
